# Adherence to the Mediterranean Diet and Academic Performance in University Students: A Systematic Review and Meta-Analysis

**DOI:** 10.3390/nu18172866

**Published:** 2026-09-02

**Authors:** Fernando Peral-Martínez, Bruno Bizzozero-Peroni, Valentina Díaz-Goñi, Tomás Olivo-Martins-de-Passos, Ana María Peraile-Huerta, Alejandro Prieto-Ayuso, Estela Jiménez-López, Vicente Martínez-Vizcaíno, Arthur Eumann Mesas

**Affiliations:** 1 Health and Social Research Center, Universidad de Castilla-La Mancha, 16071 Cuenca, Spain; fernando.peral@uclm.es (F.P.-M.); tomas.olivo@uclm.es (T.O.-M.-d.-P.); anamaria.peraile@alu.uclm.es (A.M.P.-H.); estela.jimenezlopez@uclm.es (E.J.-L.); vicente.martinez@uclm.es (V.M.-V.); arthur.emesas@uclm.es (A.E.M.); 2 Health Research Institute of Castilla-La Mancha (IDISCAM), 45071 Toledo, Spain; 3 Faculty of Education, Universidad de Castilla-La Mancha, 13071 Ciudad Real, Spain; 4 Higher Institute of Physical Education, Universidad de la República, Maldonado 20000, Uruguay; 5 Aging Research Center, Department of Neurobiology, Care Sciences and Society, Karolinska Institutet and Stockholm University, SE-17177 Stockholm, Sweden; 6 Department of Physical Education, Arts Education, and Music, Faculty of Education, Universidad de Castilla-La Mancha, 16071 Cuenca, Spain; alejandro.prieto@uclm.es; 7 Centro de Investigación Biomédica en Red de Salud Mental (CIBERSAM), Instituto de Salud Carlos III, 28029 Madrid, Spain; 8 Faculty of Health Sciences, Universidad Autónoma de Chile, Talca 3465548, Chile

**Keywords:** dietary patterns, healthy eating, academic achievement, educational outcomes, young adult

## Abstract

**Background/Objectives**: Adherence to the Mediterranean diet (MedDiet) has been associated with numerous health benefits, yet it is often suboptimal among university students. Its relationship with academic performance (AP), an outcome with potential long-term implications, has not been quantitatively synthesized in this population. This study aimed to synthesize the available evidence on the associations between MedDiet adherence and AP in university students. **Methods**: A systematic review and meta-analysis followed PRISMA 2020 and MOOSE guidelines (PROSPERO: CRD42024526903). PubMed, Web of Science, Scopus, and the Cochrane Library were searched from inception to 6 August 2026. AP was examined through objective (grade point average, mean academic grade, or university entrance scores) and subjective measures (academic self-concept or perceived academic progress). Standardized mean differences (SMDs) were pooled using random-effects models. Subgroup, meta-regression, and sensitivity analyses were performed. Methodological quality was appraised with the NIH tool, and certainty of evidence with GRADE. **Results**: Twelve cross-sectional studies were included (10,931 students; study-level mean ages: 19.0–25.1 years; 56.7% female). A positive association was observed in the primary analysis of objective AP measures (10 studies; SMD = 0.49; 95% CI: 0.04 to 0.94; *I*^2^ = 98.2%) and for subjective measures (3 studies; SMD = 0.42; 95% CI: 0.14 to 0.71; *I*^2^ = 0.0%). The certainty of the evidence was very low. **Conclusions**: Higher MedDiet adherence may be associated with better AP in university students. However, considerable between-study heterogeneity and very low certainty of the evidence limit confidence in the pooled estimates. Further high-quality longitudinal and intervention studies are needed.

## 1. Introduction

The Mediterranean diet (MedDiet) is a predominantly plant-based dietary pattern traditionally followed in countries bordering the Mediterranean Sea and recognized by UNESCO as part of the Intangible Cultural Heritage of Humanity [1]. It is widely regarded as one of the most extensively studied models of healthy eating [2]. This pattern is characterized by a high intake of fruits, vegetables, whole grains, legumes, and nuts; a moderate consumption of fish and dairy products; and a limited intake of red and processed meat, with olive oil serving as the principal source of dietary fat [3]. This food combination provides a nutritional profile particularly rich in monounsaturated fatty acids, dietary fiber, and polyphenols [4]. Owing to this favorable composition, adherence to the MedDiet has attracted substantial scientific interest over recent decades, driven by its consistent association with a wide range of health benefits [2].

Among these benefits, the strongest evidence concerns the prevention of major chronic diseases, although its potential influence may extend to other health domains. An umbrella review of meta-analyses of observational studies and randomized controlled trials, including 37 health outcomes across more than 12.8 million participants, reported that higher MedDiet adherence was associated with a reduced risk of overall mortality, cardiovascular disease, cancer incidence, neurodegenerative disease, and diabetes [5]. The same dietary components underlying these associations may also influence brain health, given the antioxidant, anti-inflammatory, and neuroprotective properties of the MedDiet’s main constituents, such as monounsaturated and omega-3 fatty acids, polyphenols, and vitamins [6,7,8]. Accordingly, higher MedDiet adherence has been associated with better mental and cognitive health, including a lower risk of depression and cognitive impairment [9,10,11,12,13].

These associations may be particularly relevant during early adulthood, when brain maturation remains ongoing [14] and dietary behaviors are still being established [15]. For many students, this stage coincides with the transition to university, which can involve substantial lifestyle adjustments, particularly for those who move away from the family home, and has been associated with reduced diet quality and physical activity [16]. In this context, MedDiet adherence is frequently suboptimal among young people. In Spain, only around 38% show high adherence, with a marked decline over the last decade [17,18]. In higher education, a multicenter study found that only 23.6% of first-year Spanish students met the criterion for good MedDiet adherence [19]. Adherence in this population may also be shaped by socioeconomic and behavioral factors. Limited financial resources and food insecurity may restrict access to foods characteristic of the MedDiet [20], while skipping breakfast and smoking have been associated with lower adherence [21]. However, whether the association between MedDiet adherence and academic performance (AP) is independent of these socioeconomic and behavioral factors remains unclear. Given that adequate nutrition may support the cognitive processes involved in learning [8,22] and that these processes are relevant to AP [23], lower MedDiet adherence could plausibly be associated with poorer AP, warranting investigation in this population.

Within this context, AP among university students is an important educational outcome, given its associations with cognitive and mental health [24,25] and life satisfaction [26], as well as its potential contribution to employment and socioeconomic prospects later in life [27]. AP is a complex construct, influenced by factors such as prior knowledge, intelligence, and motivation [28]. It generally refers to achievement in academic tasks, commonly assessed through course grades, examination results, cumulative grade point average (GPA), or university entrance grades [29]. These objective measures should be distinguished from academic self-concept, which reflects students’ perceptions of their academic competence rather than their demonstrated achievement. Although related, these outcomes are conceptually distinct constructs that do not necessarily converge, given that self-perceived competence does not always align with objectively measured achievement [30]. Nevertheless, the two perspectives may be complementary, as subjective constructs reflect aspects of students’ academic experience not fully captured by objective AP indicators. Examining both may therefore provide a more complete picture of the potential association between MedDiet adherence and AP at the university level.

To date, evidence on the association between MedDiet adherence and AP has focused mainly on children and adolescents, in whom a recent systematic review and meta-analysis of 18 studies reported a significant, though weak, positive association [31]. In university students, the evidence is considerably more limited. A narrative review published in 2020 identified only two studies examining this association and highlighted the scarcity of evidence in this population [32]. The COVID-19 pandemic further underscored the relevance of this question: during distance education, changes in study context, impaired concentration, and psychological distress were associated with poorer self-reported AP [24,33], while pandemic-related restrictions also affected students’ food environments and eating behaviors [34]. Together, these observations illustrate how contextual disruptions can affect both AP and diet, a potentially modifiable lifestyle factor. Nevertheless, to the best of our knowledge, no previous systematic review and meta-analysis has synthesized this association specifically in university students.

To address this gap, the main objective of the present systematic review and meta-analysis was to synthesize the available evidence on the associations between MedDiet adherence and AP in university students, as measured objectively through mean academic grade, GPA, and university entrance scores. Additional exploratory analyses were conducted to synthesize the evidence based on subjective measures of AP, specifically academic self-concept and perceived academic progress.

## 2. Materials and Methods

This systematic review and meta-analysis was reported in accordance with the Preferred Reporting Items for Systematic Reviews and Meta-Analyses (PRISMA) 2020 statement [35] (see Table S1 in the Supplementary Materials) and the Meta-analysis of Observational Studies in Epidemiology (MOOSE) guidelines [36] (Table S2). The review protocol was registered in PROSPERO (registration number: CRD42024526903). Two reviewers (F.P.-M. and B.B.-P.) independently performed the literature search, study selection, data extraction, and methodological quality assessment. Disagreements were resolved by consensus, with a third researcher (A.E.M.) acting as decision-maker.

### 2.1. Data Sources and Search Strategy

Systematic searches were conducted in the following electronic databases from inception to 23 September 2025: PubMed, Web of Science, Scopus, and the Cochrane Library. On the same date, supplementary searches were performed in Google Scholar. No restrictions on publication date or language were applied. The search was updated on 6 August 2026, using the same sources and search terms but restricted to records published between 23 September 2025 and 6 August 2026. Reference lists of the included studies and relevant reviews were also screened throughout the review process to identify additional records. Search terms were combined with Boolean operators and structured around three conceptual blocks: Mediterranean diet, academic performance, and university students. The full search strategy for each source is reported in Table S3, including the exact string as implemented, the dates of the initial and updated searches, and the number of records retrieved.

### 2.2. Eligibility Criteria

Eligibility criteria were defined according to the Population, Exposure, Comparison, Outcome, and Study design (PECOS) framework and are detailed in Table 1. For studies with mixed populations, inclusion required that data for university students be separately extractable.

Studies were excluded if they (1) were reviews, editorials, protocols, non-peer-reviewed reports, or animal studies; (2) assessed diet as the intake of single nutrients, foods, or food groups rather than overall MedDiet adherence; (3) did not report quantitative data on the association between MedDiet adherence and AP; or (4) were based on duplicate or overlapping samples. When multiple reports shared an overlapping sample, they were treated as a single study, retaining the report with the most complete data. When relevant data were missing or inconsistent, the corresponding authors were contacted by email.

### 2.3. Study Selection

All records were imported into the Rayyan systematic review web application [37], where duplicates were removed. A two-stage screening process was then applied. First, based on title and abstract, studies that clearly did not address the association between MedDiet adherence and AP in university students were excluded. Second, the remaining studies were analyzed by reading the full text to determine whether they met the eligibility criteria. Disagreements at either stage were resolved through discussion, with adjudication by the third reviewer when required (A.E.M.).

### 2.4. Data Extraction

Data were extracted using a pre-designed spreadsheet developed by the research team. The following information was collected from each study: (1) authors and year of publication; (2) country in which data were collected; (3) study design; (4) sample size; (5) participant information (proportion of female, age, body mass index [BMI]); (6) the instrument used to assess MedDiet adherence and its categorization; (7) the measure of AP (objective and/or subjective) and its categorization; (8) methodological quality characteristics; and (9) the main findings, including the effect size estimates, the statistical data required to compute or convert them (e.g., means and standard deviations, correlation coefficients, and category-specific frequencies), and covariate adjustments. When a study reported eligible outcomes of more than one type, each was extracted and assigned to the corresponding objective or subjective category. When both unadjusted and adjusted estimates were available, the most fully adjusted model was used.

### 2.5. Methodological Quality Assessment

The methodological quality of the included studies was assessed using the National Institutes of Health (NIH) Quality Assessment Tool for Observational Cohort and Cross-Sectional Studies [38]. This tool comprises 14 items covering domains such as the study population, exposure and outcome measures, and statistical analyses. Each item was rated as “yes” when the criterion was met, “no” when it was not, “not reported” when the required information was not provided, or "cannot determine" when the available information was insufficient to make a judgment. Items 6, 7, and 13, which concern temporality and follow-up, were considered not applicable to cross-sectional designs. These ratings contributed to an overall judgment, and each study was classified as good (most criteria met), fair (some criteria met), or poor (few criteria met) according to the NIH quality rating guide. Because the eligibility criteria permitted experimental designs, randomized controlled trials would be assessed using the revised Cochrane risk-of-bias tool for randomized trials (RoB 2) [39], whereas non-randomized studies of interventions would be assessed using the Risk Of Bias In Non-randomized Studies of Interventions (ROBINS-I) tool [40].

### 2.6. Statistical Analysis

Descriptive analyses were performed to summarize the characteristics of the included studies and their participants. Categorical variables were reported as absolute frequencies and percentages. Continuous variables were presented using the summary measures reported by the primary studies, generally means and standard deviations, and ranges of study-level means were used to summarize variation across studies when applicable.

#### 2.6.1. Exposure Harmonization

MedDiet adherence was analyzed according to whether it was reported as a categorical or continuous exposure. For categorical exposures, the highest adherence category was compared against the lowest, which served as the reference. When no participants reached the highest predefined category, the highest available category was used as the upper contrast [41]. For studies reporting MedDiet adherence as a continuous score, effect sizes were derived from the corresponding continuous association when no categorical contrast was available. Because the MedDiet adherence instruments differed in their score ranges and cut-off points, adherence categories were defined by the thresholds reported in each original study rather than by a common threshold.

#### 2.6.2. Effect Sizes

The standardized mean difference (SMD), expressed as Cohen’s *d* with its 95% confidence interval (CI), was used as the common effect size for all analyses. All estimates were oriented with MedDiet adherence as the exposure and AP as the outcome, so that positive values indicated better AP with higher MedDiet adherence. Covariate-adjusted estimates were used preferentially when available in this direction. Otherwise, estimates were derived from the available unadjusted data. Depending on the data reported by each study, Cohen’s *d* and its standard error were derived from one of three types of input data: group means and standard deviations, odds ratios (ORs) computed from 2 × 2 category frequencies, or correlation coefficients. These estimates were converted to SMDs by applying the appropriate formula [42,43,44,45]. All conversions were performed using Comprehensive Meta-Analysis (CMA) version 4 (Biostat Inc., Englewood, NJ, USA) [46].

#### 2.6.3. Data Synthesis

Separate meta-analyses were conducted for objective (primary analysis) and subjective (exploratory analysis) AP measures. Because one study reported both types of outcome, it was included in each of the corresponding analyses [47]. Effect sizes were pooled using a random-effects model with the DerSimonian-Laird estimator [48]. Statistical heterogeneity was assessed using the *I*^2^ statistic, classified as not important (0–40%), moderate (30–60%), substantial (50–90%), or considerable (75–100%) [49], along with the corresponding *p*-value. A 95% prediction interval was calculated to reflect the expected range of true effects in future studies [50].

#### 2.6.4. Additional Analyses

Several complementary analyses were performed for the primary meta-analysis (objective AP). First, exploratory subgroup analyses were conducted according to geographic region (Europe versus Asia) and to the MedDiet adherence instrument used in the included studies (Mediterranean Diet Adherence Screener [MEDAS-14] versus Mediterranean Diet Quality Index in Children and Adolescents [KIDMED]), with differences between subgroups tested. Second, random-effects meta-regressions were conducted to examine three study-level moderators: proportion of women, mean age, and mean BMI. Given that fewer than 10 studies contributed to some meta-regression models [49], these analyses were considered exploratory and should be interpreted with caution. Third, the robustness of the pooled estimate was assessed through several sensitivity analyses: re-estimation of the effect size using Hedges’ *g* to correct for small-sample bias [51]; a leave-one-out approach, in which the pooled estimate was recalculated after sequentially omitting each study; and restriction to studies conducted in Mediterranean countries. Small-study effects and potential publication bias were evaluated by visual inspection of a funnel plot and Egger’s regression test [52].

Statistical significance was set at *p* < 0.05. All analyses were conducted using R (version 4.5.2; R Foundation for Statistical Computing, Vienna, Austria) with the meta [53] and metafor [54] packages.

### 2.7. Certainty of Evidence

The Grading of Recommendations Assessment, Development and Evaluation (GRADE) approach was used to determine the certainty of the evidence for the primary meta-analysis [55], using GRADEpro GDT software [56]. Evidence from observational studies begins at a low level of certainty and may be rated down across five domains (risk of bias, inconsistency, indirectness, imprecision, and publication bias) or rated up when a large effect, a dose–response gradient, or residual confounding acting against the observed association is present. The overall certainty of the evidence was accordingly rated as high, moderate, low, or very low [57].

## 3. Results

### 3.1. Study Selection

**Figure 1:**
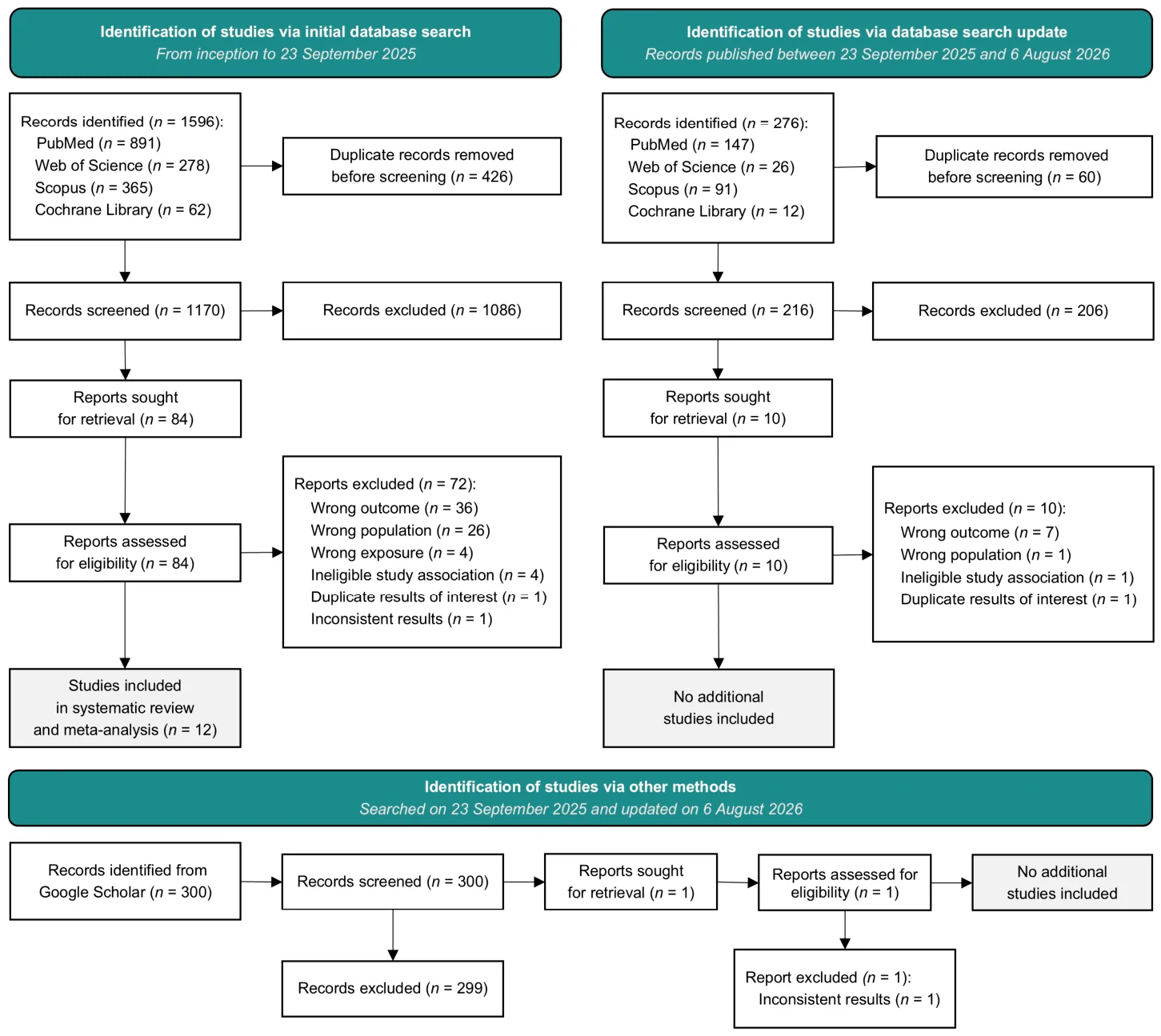
PRISMA 2020 flow diagram of study selection.

The systematic search identified a total of 1872 records across the four databases and 300 additional records from Google Scholar. After removing duplicates, 1686 records were screened by title and abstract, and 95 reports were assessed for eligibility in full text. Of these, 83 were excluded for various reasons (Table S4), including two studies excluded due to unresolved data inconsistencies after the corresponding authors were contacted for clarification [58,59]. Thus, 12 studies met the eligibility criteria and were included in the systematic review and meta-analysis [19,20,41,47,60,61,62,63,64,65,66,67]. Neither the database search update nor the supplementary searches yielded additional studies for inclusion. Figure 1 shows the study selection process.

### 3.2. Study Characteristics

Table 2 and Table S5 summarize the main characteristics of the included studies. All 12 studies had a cross-sectional design and were published between 2017 [65] and 2025 [61,66].

#### 3.2.1. Population

The studies included a total of 10,931 university students. Among the 10 studies reporting sex distribution [19,41,47,60,61,62,63,65,66,67], 56.7% of participants were females. The mean age of participants ranged from 19.0 [67] to 25.1 [47] years. BMI was reported in eight of the 12 studies, with mean values ranging from 21.9 [63] to 24.0 [60] kg/m^2^. The studies were conducted in Spain [19,41,47,64,67], Türkiye [20,65], Greece [61], Lebanon [62], Italy [63], and the United Arab Emirates [60], with one additional multinational study across Slovenia, Croatia, and Montenegro [66].

#### 3.2.2. Exposure and Outcome

MedDiet adherence was assessed using MEDAS-14 in five studies [19,20,41,47,63] and KIDMED in seven studies [60,61,62,64,65,66,67]. Across the nine studies reporting the distribution of adherence categories [19,41,47,60,61,62,63,65,67], eight reported the highest category separately, whereas Naja et al. [60] reported low versus combined medium-to-high adherence. In these eight studies, the proportion of students in the highest adherence category ranged widely, from 0.0% [41] to 77.6% [67].

AP was assessed using objective and subjective measures. Objective measures, based on students’ academic grades, were the mean academic grade [41,47,61,64,66], the GPA [20,60,62,65], and the university entrance examination (UEE) score [19]. The mean academic grade corresponds to the average mark recorded in the student’s academic transcript. The GPA is a numerical summary of AP obtained by converting course grades to points and averaging them, reported on a 0–4 scale. The UEE score is the standardized mark used for university admission in Spain, which combines the average grade obtained over the two years of upper-secondary education (or an equivalent vocational program) with the score achieved in the entrance examination. Subjective measures, based on self-reported validated scales, were academic self-concept [47,67] and perceived academic progress [63]. Academic self-concept reflects students’ own perception of their AP and was assessed with the academic subscale of the Form-5 Self-Concept Questionnaire (AF-5) [68]. Perceived academic progress captures students’ subjective appraisal of their own academic advancement. One study reported both objective and subjective measures [47].

### 3.3. Quality Assessment

Given that all included studies had a cross-sectional design, only the NIH Quality Assessment Tool [38] was applied. Based on this assessment, 2 studies (16.7%) were rated as good quality [19,61] and 10 (83.3%) as fair quality [20,41,47,60,62,63,64,65,66,67]. The criteria most frequently unmet were: (i) the repeated assessment of the exposure over time, (ii) the blinding of outcome assessors to participants’ exposure status, (iii) adjustment for confounders, (iv) the reporting of a participation rate of at least 50%, and (v) the justification of the sample size. Detailed ratings for each study are provided in Table S6.

### 3.4. Narrative Synthesis of Study Aims, Methodological Approaches, and Findings

The included studies varied in how directly they examined the association between MedDiet adherence and AP. Sample sizes and recruitment scope also varied substantially, ranging from 55 participants at a single institution [64] to 5433 students recruited across ten Greek regions [61], with several studies using multicampus, multiregional, or multinational samples [19,61,66,67]. Alfaro-González et al. (2024) [19] was the only study whose stated aims focused exclusively on this relationship, examining both the overall MEDAS-14 score and its individual items in relation to university entrance scores. Five studies included objective AP as a stated component of broader aims concerning diet quality, breakfast habits, and anthropometric measures [65]; healthy lifestyle behaviors [41]; MedDiet adherence and physical activity [64]; diet, physical activity, anxiety, and self-concept [47]; or factors associated with MedDiet adherence, including academic achievement [62]. In four studies, objective AP was a secondary variable within broader investigations of food insecurity and psychosocial health [20], perceived stress and sleep quality [61], sleep quality and chronotype [60], or MedDiet knowledge and adherence [66]. Three studies examined subjective AP, specifically academic self-concept [47,67] or perceived academic progress [63], with Melguizo-Ibáñez et al. (2022) [47] contributing both objective and subjective AP outcomes.

The evidence was homogeneous in study design but heterogeneous in the assessment of both MedDiet adherence and AP. All studies were cross-sectional and measured exposure and outcome at a single time point. MedDiet adherence was assessed using either MEDAS-14 or KIDMED, with study-specific categorizations and cut-off points (Table 2). No study derived the MedDiet adherence exposure from a comprehensive dietary assessment such as a food frequency questionnaire or dietary record, although Alfaro-González et al. (2024) [19] used a food frequency questionnaire to estimate total energy intake as a covariate. Objective AP included university entrance scores, mean course grades, or GPA, which were analyzed as continuous scores or categories and were self-reported in several studies.

The available data consisted of group comparisons [19,47,65,67], bivariate correlations [20,47,63,64,66], or cross-tabulations of MedDiet adherence and AP categories [41,60,61,62]. Only Alfaro-González et al. (2024) [19] provided an estimate adjusted for a prespecified set of potential confounders. The remaining estimates were not adjusted for potential confounders of the MedDiet–AP association, and the adjusted model reported by Dakanalis et al. (2025) [61] examined the association in the opposite direction. All extracted estimates were therefore oriented consistently, with MedDiet adherence as the exposure and AP as the outcome.

For objective AP, the original reports converged in direction but not in statistical support. Five studies reported statistically significant positive associations [19,47,61,62,65], whereas the other five reported positive but non-significant associations [20,41,60,64,66]. These results correspond to the analyses reported in the original articles. In El Hajj and Julien (2021) [62], the overall association was statistically significant, whereas the extreme-category contrast extracted for the meta-analysis included fewer participants and was imprecise. After harmonization, all ten study-specific estimates indicated better AP with greater MedDiet adherence, although their magnitudes varied markedly. The largest estimate was derived from the extreme-category comparison in Dakanalis et al. (2025) [61], whereas those from Esin and Ayyıldız (2024) [20], Naja et al. (2022) [60], and Vujačić et al. (2025) [66] were close to the null. This variation cannot be attributed to a single methodological factor because the adherence instrument, AP measure, sample characteristics, and analytical contrast varied together across studies.

Evidence concerning subjective AP was more limited. Gianfredi et al. (2018) [63] reported a significant positive correlation between MedDiet adherence and perceived academic progress. The original analyses also identified differences in academic self-concept according to adherence level [47,67], although the high-versus-low contrasts extracted for the present review were imprecise. All three estimates indicated a positive direction, but the small number of studies and the use of only two subjective constructs limit conclusions regarding consistency.

Several areas remained supported by little or no evidence. No longitudinal or intervention study met the eligibility criteria. Geographic coverage was concentrated in Mediterranean settings, with Naja et al. (2022) [60] being the only study conducted outside this region. Thus, the literature converged on a positive cross-sectional direction but remained inconclusive regarding the magnitude, independence from confounding, temporality, and consistency of the association across population subgroups. Detailed study-level information on adherence distributions, extracted data, computed statistics, directions of association, and adjustment sets is provided in Table S5.

### 3.5. Meta-Analysis

#### 3.5.1. Objective Academic Performance

**Figure 2:**
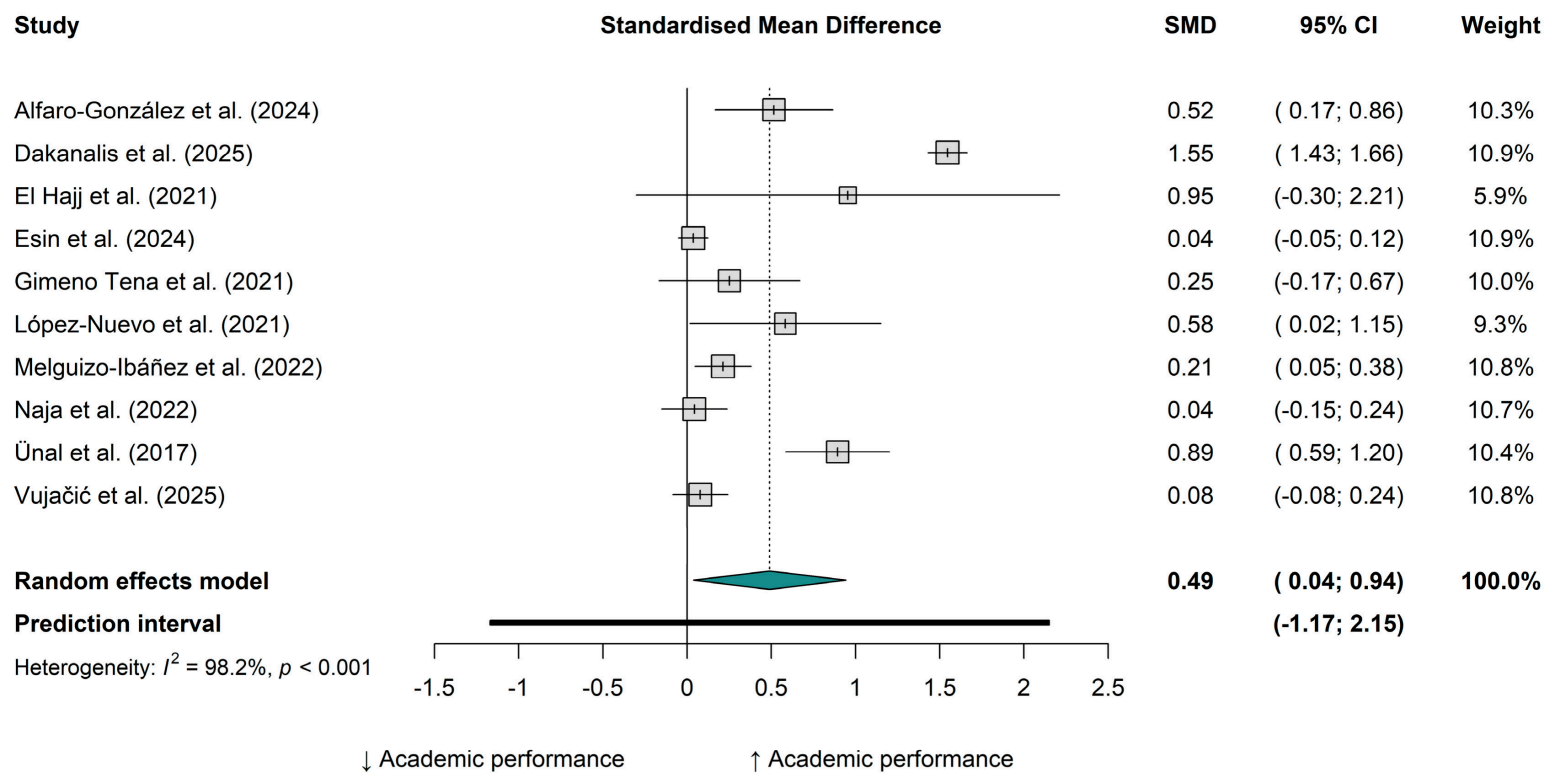
Random-effects forest plot (DerSimonian–Laird estimator) of the association between Mediterranean diet adherence and objective academic performance, expressed as the standardized mean difference (Cohen’s *d*), with 95% confidence and prediction intervals [19,20,41,47,60,61,62,64,65,66]. Positive SMD values indicate better AP with higher MedDiet adherence. Abbreviations: AP, academic performance; CI, confidence interval; MedDiet, Mediterranean diet; SMD, standardized mean difference.

Higher MedDiet adherence was significantly associated with better objective AP (SMD = 0.49; 95% CI: 0.04 to 0.94; *p* = 0.034), based on 10 studies including 10,217 university students [19,20,41,47,60,61,62,64,65,66] (Figure 2). Although all observed study-specific estimates were in the positive direction (SMD range, 0.04 to 1.55), between-study heterogeneity was considerable (*I*^2^ = 98.2%; *p* < 0.001), and the prediction interval was wide and crossed the null (−1.17 to 2.15).

#### 3.5.2. Subjective Academic Performance

**Figure 3:**
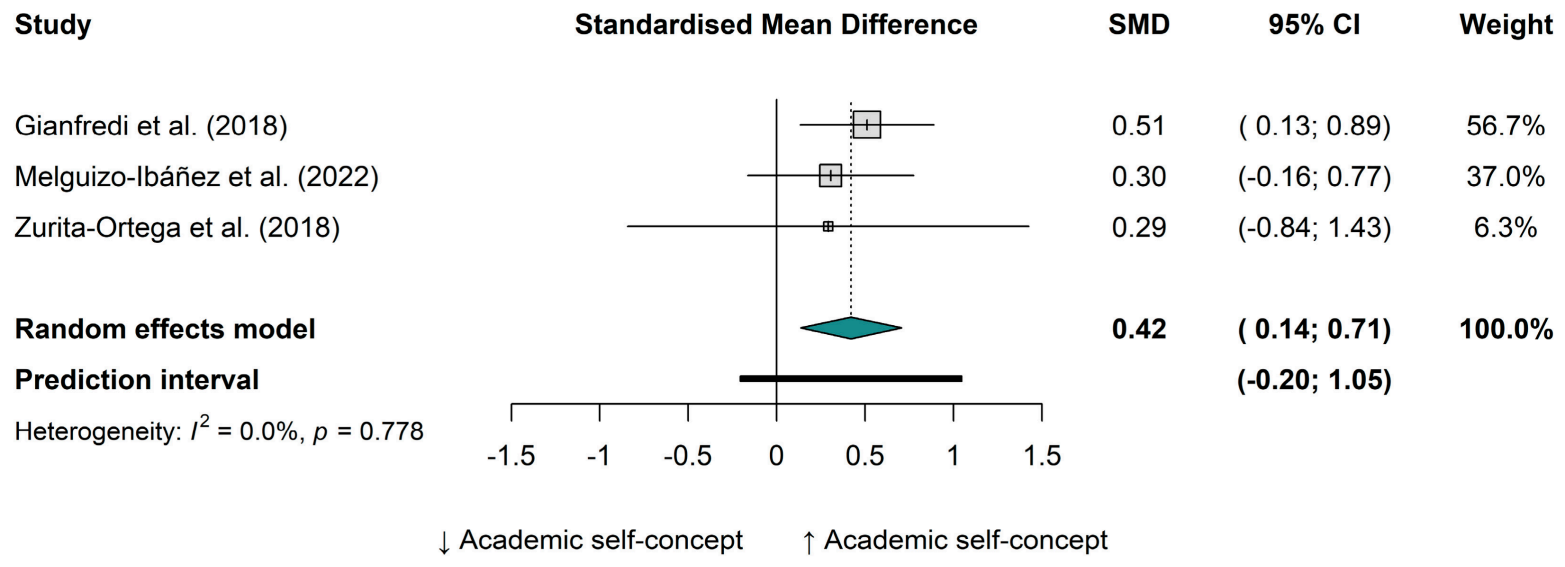
Random-effects forest plot (DerSimonian–Laird estimator) of the association between Mediterranean diet adherence and subjective academic performance, expressed as the standardized mean difference (Cohen’s *d*), with 95% confidence and prediction intervals [47,63,67]. Positive SMD values indicate better subjective AP (A-SC or perceived academic progress) with higher MedDiet adherence. Abbreviations: AP, academic performance; A-SC, academic self-concept; CI, confidence interval; MedDiet, Mediterranean diet; SMD, standardized mean difference.

Higher MedDiet adherence was also significantly associated with better subjective AP (SMD = 0.42; 95% CI: 0.14 to 0.71; *p* = 0.004), based on 3 studies comprising 1272 university students [47,63,67] (Figure 3). No heterogeneity was observed (*I*^2^ = 0.0%; *p* = 0.778), and the 95% prediction interval ranged from −0.20 to 1.05.

### 3.6. Subgroup Analyses and Meta-Regressions

Subgroup and meta-regression analyses were conducted for the primary analysis (objective AP). In the subgroup analyses by geographic region, the association between higher MedDiet adherence and better objective AP did not reach statistical significance in either European or Asian studies, with no significant difference between subgroups (*p* = 0.626; Figure S1). When studies were stratified by MedDiet adherence instrument, the association remained significant among studies using the MEDAS-14 (SMD = 0.20; 95% CI: 0.01 to 0.39; *I*^2^ = 69.5%; prediction interval: −0.37 to 0.77) but not among those using the KIDMED (SMD = 0.67; 95% CI: −0.05 to 1.39; *I*^2^ = 98.3%; prediction interval: −1.73 to 3.06), although the difference between subgroups was not significant (*p* = 0.219; Figure S2). Meta-regression models showed that none of the participant characteristics considered (proportion of women, mean age, or mean BMI) significantly influenced the association between MedDiet adherence and objective AP (*p* = 0.794, 0.691, and 0.545, respectively; Figure S3).

### 3.7. Sensitivity Analyses

Several sensitivity analyses were conducted to test the robustness of the primary estimate. The pooled estimate remained significant after recalculating effect sizes as Hedges’ *g* to correct for potential small-sample bias (SMD = 0.49; 95% CI: 0.04 to 0.94; Figure S4). In the leave-one-out analysis, the pooled estimate ranged from 0.29 (omitting Dakanalis et al. [61]) to 0.55 (omitting Esin et al. [20]). Statistical significance was lost when Ünal et al. [65] or El Hajj et al. [62] were removed (Figure S5). The association remained significant when the analysis was restricted to studies conducted in Mediterranean countries, excluding the only study from a non-Mediterranean country [60] (SMD = 0.54; 95% CI: 0.05 to 1.04; Figure S6). According to Egger’s regression test and funnel plot inspection, there was no evidence of publication bias (*p* = 0.890; Figure S7).

### 3.8. Certainty of Evidence

According to the GRADE assessment, the certainty of evidence for the association between MedDiet adherence and objective AP was rated as very low. The evidence was downgraded for serious risk of bias, very serious inconsistency, very serious indirectness, and serious imprecision. Full details are provided in Table S7.

## 4. Discussion

To the best of our knowledge, this is the first systematic review and meta-analysis to examine the association between MedDiet adherence and AP in university students. Higher MedDiet adherence may be associated with better AP, based on both objective and subjective measures, with pooled estimates in the small-to-moderate range. However, these findings should be interpreted with caution given the limitations of the available evidence, including the cross-sectional design of all included studies, considerable heterogeneity in the primary analysis, and the very low certainty of the evidence. Accordingly, the pooled estimates should be interpreted as reflecting a possible cross-sectional association rather than as evidence that MedDiet adherence improves AP.

Our findings are consistent with the broader literature on diet quality and AP in university students. Phelan et al. [69], in a systematic review of college students, reported weak positive associations between overall diet quality and GPA in the two studies evaluating this outcome, although these used validated diet quality indices rather than MedDiet-specific instruments. Although these are distinct constructs, validated diet quality indices generally reward greater intake of fruits, vegetables, whole grains, legumes, and nuts [70], which are also key components of the MedDiet [3]. This shared emphasis on nutrient-dense plant foods provides a plausible basis for the consistent direction of these findings and supports further examination of the MedDiet as a specific dietary pattern. Focusing on the MedDiet specifically, Antonopoulou et al. [32], in a 2020 narrative review, identified only two studies [63,67] examining the association between MedDiet adherence and AP in university students, both assessing subjective AP. These two studies were included in the present subjective-outcome meta-analysis together with one additional study [47]. The present review therefore expands the previous literature by quantitatively synthesizing studies of both objectively measured AP and subjective academic constructs. Nevertheless, these outcome categories should remain conceptually separate because academic self-concept or perceived progress cannot be assumed to measure the same construct as grades or GPA.

In the absence of an equivalent synthesis specifically focused on university students, two meta-analyses conducted in younger populations provide limited contextual evidence. López-Gil et al. [31] reported a small positive association between MedDiet adherence and AP in children and adolescents, while Treister-Goltzman and Peleg [71] similarly reported a positive association with academic achievement in school-aged populations. Although these findings are consistent in direction with our results, differences in developmental stage, educational setting, outcome assessment, and effect measures preclude direct comparison with our estimate or extrapolation to university students.

The considerable heterogeneity observed in our study for objective AP reflected differences in the magnitude rather than the direction of the individual estimates. All ten studies reported positive effect estimates, yet these ranged widely (SMD range: 0.04 to 1.55). However, the consistency in the observed direction should not be overinterpreted because the 95% prediction interval ranged from −1.17 to 2.15 and crossed the null, indicating that the true association in a future comparable study could plausibly be negative, null, or positive. This prediction interval limits the generalizability of the average pooled estimate.

To explore potential sources of heterogeneity, we examined several study-level characteristics. In subgroup analyses, the association remained significant only among studies using the MEDAS-14, which yielded a smaller estimate with lower heterogeneity than the KIDMED. One possible explanation is that the MEDAS-14 was originally developed and validated for adults [72], whereas the KIDMED was developed using data from children and youths aged 2–24 years [73]. Although subsequent studies have provided evidence of moderate-to-good test–retest reliability and reasonable validity in university samples [74,75], this evidence remains limited, and neither study assessed criterion validity against a detailed dietary assessment. The KIDMED may therefore capture adult dietary patterns less precisely than adult-oriented instruments, potentially contributing to the greater heterogeneity observed among KIDMED-based studies. However, the difference between instrument subgroups was not statistically significant, so this explanation remains tentative. Differences in the measurement of objective AP may also have contributed to the considerable heterogeneity, as it was assessed with different instruments and grading scales (mean academic grade, GPA, and university entrance examination scores). In contrast, no statistical heterogeneity was observed in the subjective AP analysis (*I*^2^ = 0.0%), although this result is difficult to interpret given that it was based on only three studies. The influence of individual studies should also be considered, as omitting Dakanalis et al. [61] reduced the heterogeneity, suggesting that some large studies may have contributed disproportionately to the observed variability. In addition, geographic region did not account for the heterogeneity in subgroup analysis, nor did the proportion of women, mean age, or mean BMI in the meta-regression models.

Several biological pathways could contribute to the association between MedDiet adherence and AP, potentially through improvements in cognitive function [6,22]. Components of the MedDiet, including olive oil, nuts, fish, fruits, and vegetables, provide monounsaturated and omega-3 fatty acids, polyphenols, fiber, vitamins, and minerals that may influence oxidative stress, inflammation, neuronal membrane function, and synaptic plasticity [8,76,77,78]. The MedDiet may also influence communication along the microbiota–gut–brain axis through effects on the composition and function of the gut microbiota [79]. These mechanisms are biologically plausible but were not directly tested in the included studies, nor was cognitive function established as a mediator between MedDiet adherence and AP. Consequently, the biological mechanisms discussed here should be regarded as hypotheses rather than as explanations demonstrated by the present meta-analysis.

Beyond the biological pathways, MedDiet adherence may also operate through behavioral mechanisms: higher adherence has been associated with better sleep quality and lower perceived stress, both of which are in turn related to AP [80] and are particularly relevant during university, a stage characterized by irregular eating, disrupted sleep, and high academic demands [16]. Nonetheless, sleep quality, stress, physical activity, alcohol use, socioeconomic status, food security, health literacy, motivation, and self-regulation may operate as confounders, mediators, or both [20,80,81,82]. For example, students with greater socioeconomic resources may simultaneously exhibit healthier diets and better academic outcomes [20]. Because all included studies were cross-sectional, reverse causation cannot be excluded; higher-achieving students might adopt healthier dietary patterns, possibly reflecting greater health awareness or more regular routines, while academic stress and workload may likewise affect dietary behavior [83].

In addition to the issues discussed above, several methodological considerations should be considered when interpreting these findings. First, only one study provided a covariate-adjusted estimate for the association of interest [19], whereas the remaining studies contributed unadjusted estimates. Therefore, residual confounding by factors such as socioeconomic status, physical activity, or sleep quality cannot be excluded. Second, effect sizes were derived from different data inputs (extreme-category contrasts and full-range correlations), which are not fully comparable and may have increased the observed heterogeneity. Third, the pooled estimate was sensitive to individual studies, as the leave-one-out analysis showed that omitting some studies changed the magnitude of the pooled effect and, in some cases, led to a loss of statistical significance. Fourth, several deviations from the PROSPERO-registered protocol were made before examining the outcome data, including the addition of a database, a later-than-planned search, the inability to perform the planned subgroup analyses because of insufficient data, and the addition of exploratory analyses. Finally, given the limited number of studies, the meta-analysis of subjective AP and the additional subgroup and meta-regression analyses were exploratory, and the assessment of publication bias was constrained.

This review also has several strengths. It draws on a large evidence base (10,931 participants across 12 studies; 10,217 in the primary analysis) and was conducted and reported in accordance with the PRISMA 2020 statement and MOOSE guidelines, with search strategies tailored to each database. Methodological quality and certainty of evidence were formally assessed using the NIH and GRADE tools, respectively, and the robustness of the primary estimate was examined through complementary sensitivity analyses.

The available evidence in university students shows a positive cross-sectional association, although its magnitude, temporal sequence, and independence from confounding remain uncertain. This is an emerging field, and three main priorities can be identified. First, prospective studies should assess MedDiet adherence at the beginning of an academic year and AP at its end, repeating these assessments over consecutive academic years and accounting for baseline academic attainment. Such designs would help establish the temporal sequence and reduce, although not eliminate, the risk of reverse causation, since higher-achieving students may also adopt healthier dietary patterns. If prospective evidence supports a temporally ordered association independent of major confounders, randomized trials of MedDiet-based interventions in university settings could provide stronger causal evidence. Second, more primary studies should address this association as their main research question, since it has usually been examined as one of several variables within broader investigations and reported without adjustment for potential confounders. These studies should assess MedDiet adherence using instruments validated in adults, ideally complemented by a comprehensive dietary assessment, and obtain AP from institutional records rather than from self-report, with objective and subjective outcomes reported separately. Analyses should prespecify the causal role assigned to each covariate, including socioeconomic status, food security, physical activity, alcohol use, and total energy intake, distinguishing variables treated as confounders from those evaluated as mediators. Third, studies should clarify the mechanisms and scope of the association. Candidate mediators, including cognitive function, sleep quality, and perceived stress, should be measured longitudinally. Further work should determine whether the association reflects the overall dietary pattern or particular components, whether it generalizes beyond predominantly Mediterranean settings, and whether it differs by sex or field of study.

## 5. Conclusions

This systematic review and meta-analysis suggests that higher adherence to the Mediterranean diet (MedDiet) may be associated with better academic performance (AP) in university students, based on both objective and subjective measures. However, these findings should be interpreted with caution because the pooled effect was small to moderate, between-study heterogeneity was considerable, and the certainty of the evidence was very low. Given the importance of AP and the decline in diet quality often observed during university, these results support a potential role of MedDiet adherence in AP, although they are not sufficient to inform specific dietary recommendations aimed at improving AP.

## Figures and Tables

**Table 1:** Eligibility criteria according to the PECOS framework.

Parameter	Inclusion Criterion
Population	University students, without restrictions on age, country, or field of study
Exposure	Adherence to the MedDiet assessed with a validated instrument or an established adaptation (e.g., MEDAS, KIDMED, Mediterranean Diet Score), reported as a categorical variable or as a continuous score
Comparison	Categorical exposures: highest versus lowest MedDiet adherence category defined by the original study. Continuous exposures: the change in academic performance per one-unit (or per standard-deviation) increase in the MedDiet adherence score
Outcome	AP assessed through objective measures (e.g., GPA, course or examination grades, mean academic grade, or university entrance scores) and/or subjective measures (e.g., academic self-concept or perceived academic progress)
Study design	Observational (cross-sectional, case–control, or cohort) or experimental studies

Abbreviations: AP, academic performance; GPA, grade point average; KIDMED, Mediterranean Diet Quality Index in Children and Adolescents; MEDAS, Mediterranean Diet Adherence Screener; MedDiet, Mediterranean diet; PECOS, Population, Exposure, Comparison, Outcome, and Study design.

**Table 2:** Main characteristics of the cross-sectional studies included in the systematic review and meta-analysis.

Study (Year)	Country	Sample Size (*n*)	% Female	Age, Mean ± SD (Years)	MedDiet Adherence Tool	MedDiet Adherence Categorization	AP Measure	AP Type	AP Categorization	Quality Assessment ^a^
Alfaro-González et al., 2024 [19]	Spain	266	65.4	20.8 ± 2.3	MEDAS-14 ^b^	Low (<P25) vs. high (>P75)	UEE score ^f^, z-standardized	Objective	Continuous z-score	Good
Dakanalis et al., 2025 [61]	Greece	5433	50.8	21.4 ± 2.5	KIDMED ^c^	Poor (≤3) vs. good (≥8)	Mean academic grade	Objective	Good (5.0–6.49) vs. excellent (8.5–10.0)	Good
El Hajj and Julien, 2021 [62]	Lebanon	303	69.3	Range: 18–25	KIDMED ^c^	Poor (≤3) vs. high (≥8)	GPA ^e^	Objective	Poor (<1.67) vs. excellent (>3.67)	Fair
Esin and Ayyıldız, 2024 [20]	Türkiye	2039	NR	20.9 ± 1.5	MEDAS-14 ^b^	Continuous score	GPA ^e^	Objective	Continuous score	Fair
Gianfredi et al., 2018 [63]	Italy	117	70.1	23.7 ± 4.8	MEDAS-14 ^b^	Continuous score	Perception of academic progress	Subjective	Continuous score	Fair
Gimeno Tena and Esteve Clavero, 2021 [41]	Spain	114	71.9	20.4 ± 2.6	MEDAS-14 ^b^	Low vs. medium; cut-offs NR by the authors	Mean academic grade	Objective	Below mean (≤7.37) vs. above mean (>7.37)	Fair
López-Nuevo et al., 2021 [64]	Spain	55	NR	22.0 ± 2.7 ^g^	KIDMED ^c^	Continuous score	Mean academic grade	Objective	Continuous score	Fair
Melguizo-Ibáñez et al., 2022 [47]	Spain	558	25.1	25.1 ± 6.2	MEDAS-14 ^b^	(i) Continuous score; (ii) Low (≤7) vs. optimal (>10)	(i) Mean academic grade; (ii) A-SC	(i) Objective (ii) Subjective	(i) Continuous score; (ii) Pass (5.00–6.99) vs. excellent (9.00–10.00)	Fair
Naja et al., 2022 [60]	United Arab Emirates	503	81.5	22.1 ± 4.2	KIDMED ^c^	Low (≤3) vs. high (≥4)	GPA ^e^	Objective	Low (≤3) vs. High (>3)	Fair
Ünal et al., 2017 [65]	Türkiye	365	76.4	19.7 ± 1.8	KIDMED ^c^	Low (≤3) vs. optimal (≥8)	GPA ^e^	Objective	Continuous score	Fair
Vujačić et al., 2025 [66]	Slovenia, Croatia and Montenegro	581	74.0	21.0 ± NR	KIDMED ^c^	Continuous score	Mean academic grade	Objective	Continuous score	Fair
Zurita-Ortega et al., 2018 [67]	Spain	597	73.9	19.0 ± 0.64	KIDMED ^d^	Low (≤1) vs. high (≥8)	A-SC	Subjective	Continuous score	Fair

Note: Only the categories contrasted in the present meta-analysis are shown, with cut-offs as defined by each study. For each study, the highest MedDiet adherence category was compared against the lowest; where academic performance was categorical, its highest category was likewise compared against the lowest. Abbreviations: A-SC, academic self-concept; AP, academic performance; GPA, grade point average; KIDMED, Mediterranean Diet Quality Index in Children and Adolescents; MedDiet, Mediterranean diet; MEDAS-14, 14-item Mediterranean Diet Adherence Screener; NR, not reported; SD, standard deviation; UEE, University Entrance Examination; vs, versus. ^a^ Methodological quality rated with the National Institutes of Health’s Quality Assessment Tool for Observational Cohort and Cross-Sectional studies; see Table S6 for details. ^b^ Score range 0–14. ^c^ Score range 0–12. ^d^ Score range −4 to 12. ^e^ 0–4 scale. ^f^ UEE = (0.6 × mean grade across the two years of upper-secondary education) + (0.4 × compulsory tests) + optional subjects; range 0–14; ^g^ Age was originally reported separately for two educational cycles; the two values were pooled into a single mean ± SD using the weighted mean and combined standard deviation.

## Data Availability

Data will be available upon reasonable request to the corresponding author.

## Supplementary Materials

The following supporting information can be downloaded at: https://www.mdpi.com/article/10.3390/nu18172866/s1, Table S1: PRISMA Checklist 2020; Table S2: MOOSE Checklist for Meta-analyses of Observational Studies; Table S3: Search strategy for each database; Table S4: Reason for exclusion after full-text screening; Table S5: Main findings, effect estimates, and Mediterranean diet adherence distribution of included studies; Table S6: Quality assessment of included studies; Table S7: Quality of evidence assessment according to the GRADE approach; Figure S1: Subgroup analysis of the association between Mediterranean diet adherence and objective academic performance, stratified by geographic region (Europe vs. Asia); Figure S2: Subgroup analysis of the association between Mediterranean diet adherence and objective academic performance, stratified by Mediterranean diet adherence instrument (MEDAS-14 vs. KIDMED); Figure S3: Random-effects meta-regression of the standardized mean difference for objective academic performance on three study-level moderators: proportion of women, mean age, and mean body mass index; Figure S4: Sensitivity analysis of the primary meta-analysis re-estimated using Hedges’ *g* (small-sample bias-corrected) instead of Cohen’s *d*; Figure S5: Leave-one-out sensitivity analysis of the primary meta-analysis (objective academic performance), showing the pooled random-effects estimate of the standardized mean difference recalculated after sequentially omitting each study; Figure S6: Sensitivity analysis of the primary meta-analysis (objective academic performance) restricted to studies conducted in Mediterranean countries, after excluding one non-Mediterranean study; Figure S7: Funnel plot for the assessment of small-study effects and potential publication bias in the primary meta-analysis (objective academic performance), with the standardized mean difference plotted against its standard error.

## Abbreviations

AF-5	Form-5 Self-Concept Questionnaire
AP	Academic performance
BMI	Body mass index
CI	Confidence interval
GPA	Grade point average
GRADE	Grading of Recommendations Assessment, Development and Evaluation
KIDMED	Mediterranean Diet Quality Index in Children and Adolescents
MedDiet	Mediterranean diet
MEDAS-14	14-item Mediterranean Diet Adherence Screener
MOOSE	Meta-analysis of Observational Studies in Epidemiology
NIH	National Institutes of Health
OR	Odds ratio
PECOS	Population, Exposure, Comparison, Outcome, and Study design
PRISMA	Preferred Reporting Items for Systematic Reviews and Meta-analyses
PROSPERO	International Prospective Register of Systematic Reviews
RoB 2	Risk-of-Bias tool for randomized trials
ROBINS-I	Risk of Bias in Non-randomized Studies–of Interventions
SMD	Standardized mean difference
UEE	University entrance examination
