# Supplementary material for: Adherence to the Mediterranean Diet and Academic Performance in University Students: A Systematic Review and Meta-Analysis
Source: Nutrients. 2026 Sep 2;18(17):2866. doi: 10.3390/nu18172866 (PMC13567521; doi:10.3390/nu18172866)
Supplement: Supplementary file 1 [file nutrients-18-02866-s001.zip › nutrients-4490543-Supplementary.pdf]

INDEX

Supplementary ID	Subheading	Page
**Table S1**	PRISMA Checklist 2020.	2
**Table S2**	MOOSE Checklist for Meta-analyses of Observational Studies.	5
**Table S3**	Search strategy for each database.	7
**Table S4**	Reason for exclusion after full-text screening.	8
**Table S5**	Main findings, effect estimates, and Mediterranean diet adherence distribution of included studies.	17
**Table S6**	Quality assessment of included studies.	19
**Table S7**	Quality of evidence assessment according to the GRADE approach.	20
**Figure S1**	Subgroup analysis of the association between Mediterranean diet adherence and objective academic performance, stratified by geographic region (Europe vs. Asia).	21
**Figure S2**	Subgroup analysis of the association between Mediterranean diet adherence and objective academic performance, stratified by Mediterranean diet adherence instrument (MEDAS-14 vs. KIDMED).	22
**Figure S3**	Random-effects meta-regression of the standardized mean difference for objective academic performance on three study-level moderators: proportion of women, mean age, and mean body mass index.	23
**Figure S4**	Sensitivity analysis of the primary meta-analysis re-estimated using Hedges' *g* (small-sample bias-corrected) instead of Cohen's *d*.	24
**Figure S5**	Leave-one-out sensitivity analysis of the primary meta-analysis (objective academic performance), showing the pooled random-effects estimate of the standardized mean difference recalculated after sequentially omitting each study.	25
**Figure S6**	Sensitivity analysis of the primary meta-analysis (objective academic performance) restricted to studies conducted in Mediterranean countries, after excluding one non-Mediterranean study.	26
**Figure S7**	Funnel plot for the assessment of small-study effects and potential publication bias in the primary meta-analysis (objective academic performance), with the standardized mean difference plotted against its standard error.	27

Table S1. PRISMA Checklist 2020 [1].

Section and Topic	Item #	Checklist item	Location where item is reported
**TITLE**			
Title	1	Identify the report as a systematic review.	Page 1
**ABSTRACT**			
Abstract	2	See the PRISMA 2020 for Abstracts checklist.	Page 1
**INTRODUCTION**			
Rationale	3	Describe the rationale for the review in the context of existing knowledge.	Pages 2-3
Objectives	4	Provide an explicit statement of the objective(s) or question(s) the review addresses.	Page 3
**METHODS**			
Eligibility criteria	5	Specify the inclusion and exclusion criteria for the review and how studies were grouped for the syntheses.	Page 4; Table 1
Information sources	6	Specify all databases, registers, websites, organizations, reference lists and other sources searched or consulted to identify studies. Specify the date when each source was last searched or consulted.	Page 3, Table S3
Search strategy	7	Present the full search strategies for all databases, registers and websites, including any filters and limits used.	Table S3
Selection process	8	Specify the methods used to decide whether a study met the inclusion criteria of the review, including how many reviewers screened each record and each report retrieved, whether they worked independently, and if applicable, details of automation tools used in the process.	Pages 3-4
Data collection process	9	Specify the methods used to collect data from reports, including how many reviewers collected data from each report, whether they worked independently, any processes for obtaining or confirming data from study investigators, and if applicable, details of automation tools used in the process.	Pages 3-4
Data items	10a	List and define all outcomes for which data were sought. Specify whether all results that were compatible with each outcome domain in each study were sought (e.g. for all measures, time points, analyses), and if not, the methods used to decide which results to collect.	Pages 4-6; Table 1
	10b	List and define all other variables for which data were sought (e.g. participant and intervention characteristics, funding sources). Describe any assumptions made about any missing or unclear information.	Pages 4-6

Section and Topic	Item #	Checklist item	Location where item is reported
Study risk of bias assessment	11	Specify the methods used to assess risk of bias in the included studies, including details of the tool(s) used, how many reviewers assessed each study and whether they worked independently, and if applicable, details of automation tools used in the process.	Pages 3-6; Table S6
Effect measures	12	Specify for each outcome the effect measure(s) (e.g. risk ratio, mean difference) used in the synthesis or presentation of results.	Pages 5-6; Table S5
Synthesis methods	13a	Describe the processes used to decide which studies were eligible for each synthesis (e.g. tabulating the study intervention characteristics and comparing against the planned groups for each synthesis (item #5)).	Pages 4-6
	13b	Describe any methods required to prepare the data for presentation or synthesis, such as handling of missing summary statistics, or data conversions.	Pages 5-6
	13c	Describe any methods used to tabulate or visually display results of individual studies and syntheses.	Pages 5-6; Table 2; Figures 2-3
	13d	Describe any methods used to synthesize results and provide a rationale for the choice(s). If meta-analysis was performed, describe the model(s), method(s) to identify the presence and extent of statistical heterogeneity, and software package(s) used.	Page 6
	13e	Describe any methods used to explore possible causes of heterogeneity among study results (e.g. subgroup analysis, meta-regression).	Page 6
	13f	Describe any sensitivity analyses conducted to assess robustness of the synthesized results.	Page 6
Reporting bias assessment	14	Describe any methods used to assess risk of bias due to missing results in a synthesis (arising from reporting biases).	Page 6
Certainty assessment	15	Describe any methods used to assess certainty (or confidence) in the body of evidence for an outcome.	Page 6; Table S7
**RESULTS**			
Study selection	16a	Describe the results of the search and selection process, from the number of records identified in the search to the number of studies included in the review, ideally using a flow diagram.	Pages 6-7; Figure 1
	16b	Cite studies that might appear to meet the inclusion criteria, but which were excluded, and explain why they were excluded.	Pages 6-7; Figure 1; Table S4
Study characteristics	17	Cite each included study and present its characteristics.	Pages 7-10; Table 2; Table S5
Risk of bias in studies	18	Present assessments of risk of bias for each included study.	Page 8; Table S6
Results of individual studies	19	For all outcomes, present, for each study: (a) summary statistics for each group (where appropriate) and (b) an effect estimate and its precision (e.g. confidence/credible interval), ideally using structured tables or plots.	Pages 8-13; Table 2; Table S5; Figures 2-3

Section and Topic	Item #	Checklist item	Location where item is reported
Results of syntheses	20a	For each synthesis, briefly summarise the characteristics and risk of bias among contributing studies.	Pages 8, 10; Table 2; Table S6
	20b	Present results of all statistical syntheses conducted. If meta-analysis was done, present for each the summary estimate and its precision (e.g. confidence/credible interval) and measures of statistical heterogeneity. If comparing groups, describe the direction of the effect.	Section 3.5 ; Figures 2-3
	20c	Present results of all investigations of possible causes of heterogeneity among study results.	Section 3.6; Figures S1-S3
	20d	Present results of all sensitivity analyses conducted to assess the robustness of the synthesized results.	Section 3.7; Figures S4-S6
Reporting biases	21	Present assessments of risk of bias due to missing results (arising from reporting biases) for each synthesis assessed.	Section 3.7; Figure S7
Certainty of evidence	22	Present assessments of certainty (or confidence) in the body of evidence for each outcome assessed.	Section 3.8; Table S7
**DISCUSSION**			
Discussion	23a	Provide a general interpretation of the results in the context of other evidence.	Section 4
	23b	Discuss any limitations of the evidence included in the review.	Section 4
	23c	Discuss any limitations of the review processes used.	Section 4
	23d	Discuss implications of the results for practice, policy, and future research.	Sections 4 and 5
**OTHER INFORMATION**			
Registration and protocol	24a	Provide registration information for the review, including register name and registration number, or state that the review was not registered.	Page 3
	24b	Indicate where the review protocol can be accessed, or state that a protocol was not prepared.	Page 3
	24c	Describe and explain any amendments to information provided at registration or in the protocol.	Section 4
Support	25	Describe sources of financial or non-financial support for the review, and the role of the funders or sponsors in the review.	Funding
Competing interests	26	Declare any competing interests of review authors.	Conflicts of Interest
Availability of data, code and other materials	27	Report which of the following are publicly available and where they can be found: template data collection forms; data extracted from included studies; data used for all analyses; analytic code; any other materials used in the review.	Data Availability Statement; Table S5

Table S2. MOOSE Checklist for Meta-analyses of Observational Studies [2].

Item No.	Recommendation	Reported on Page No.
**Reporting of background should include**		
1	Problem definition	2-3
2	Hypothesis statement	3
3	Description of study outcome(s)	2-4; Table 1
4	Type of exposure or intervention used	2,4; Table 1
5	Type of study designs used	4,7; Table 1
6	Study population	4,7-8; Table 1
**Reporting of search strategy should include**		
7	Qualifications of searchers (eg, librarians and investigators)	3
8	Search strategy, including time period included in the synthesis and keywords	3-4; Table S3
9	Effort to include all available studies, including contact with authors	3-4,7
10	Databases and registries searched	3; Table S3
11	Search software used, name and version, including special features used (eg, explosion)	4
12	Use of hand searching (eg, reference lists of obtained articles)	3
13	List of citations located and those excluded, including justification	6-7; Figure 1; Table S4
14	Method of addressing articles published in languages other than English	3
15	Method of handling abstracts and unpublished studies	3-4
16	Description of any contact with authors	4,7
**Reporting of methods should include**		
17	Description of relevance or appropriateness of studies assembled for assessing the hypothesis to be tested	4; Table 1
18	Rationale for the selection and coding of data (eg, sound clinical principles or convenience)	4-5
19	Documentation of how data were classified and coded (eg, multiple raters or blinding)	3-6
20	Assessment of confounding	4-5,9; Table S5
21	Assessment of study quality, including blinding of quality assessors, stratification or regression on possible predictors of study results	3-5,8; Table S6
22	Assessment of heterogeneity	6
23	Description of statistical methods (eg, complete description of fixed or random effects models, justification of whether the chosen models account for predictors of study results, dose-response models, or cumulative meta-analysis) in sufficient detail to be replicated	5-6
24	Provision of appropriate tables and graphics	Tables 1-2; Tables S1-S7; Figures 1-3; Figures S1-S7
**Reporting of results should include**		
25	Graphic summarizing individual study estimates and overall estimate	Section 3.5; Figures 2-3
26	Table giving descriptive information for each study included	Table 2; Table S5
27	Results of sensitivity testing (eg, subgroup analysis)	Sections 3.6 and 3.7; Figures S1-S6
28	Indication of statistical uncertainty of findings	Sections 3.5 and 3.6; Figures 2-3

Continued on next page …

Table S2. *Cont.*

Item No.	Recommendation	Reported on Page No.
**Reporting of discussion should include**		
29	Quantitative assessment of bias (eg, publication bias)	Section 3.7; Figure S7
30	Justification for exclusion (eg, exclusion of non-English language citations)	Section 3.1; Figure 1; Table S4
31	Assessment of quality of included studies	Sections 3.3 and 4; Table S6
**Reporting of conclusions should include**		
32	Consideration of alternative explanations for observed results	Section 4
33	Generalization of the conclusions (ie, appropriate for the data presented and within the domain of the literature review)	Sections 4 and 5
34	Guidelines for future research	Sections 4 and 5
35	Disclosure of funding source	Funding

Table S3. Search strategy for each database.

Database	Search strategy	Results (*n*)	
		Initial search	Search update
**PubMed**	(((("Mediterranean diet adherence" OR "Mediterranean scor*" OR "Mediterranean diet*" OR MedDiet OR "Mediterranean-style diet*" OR "Mediterranean eat*" OR "Mediterranean food")) AND (("academic performance" OR "academic achievement" OR learning OR educational OR intellectual OR knowledge OR excellence OR success OR "academic progress" OR "knowledge acquisition" OR accomplishment OR attainment OR "academic success"))) AND (("young adult*" OR students OR freshman OR "emerging adult*" OR college OR undergraduate*)))	891	147
**Web of Science**	TS=("Mediterranean diet adherence" OR "Mediterranean scor*" OR "Mediterranean diet*" OR MedDiet OR "Mediterranean-style diet*" OR "Mediterranean eat*" OR "Mediterranean food") AND TS=("academic performance" OR "academic achievement" OR learning OR educational OR intellectual OR knowledge OR excellence OR success OR "academic progress" OR "knowledge acquisition" OR accomplishment OR attainment OR "academic success") AND TS=("young adult*" OR students OR freshman OR "emerging adult*" OR college OR undergraduate*)	278	26
**Scopus**	TITLE-ABS-KEY(("Mediterranean diet adherence" OR "Mediterranean score" OR "Mediterranean diet" OR MedDiet OR "Mediterranean-style diet" OR "Mediterranean eating" OR "Mediterranean food")) AND TITLE-ABS-KEY(("academic performance" OR "academic achievement" OR learning OR educational OR intellectual OR knowledge OR excellence OR success OR "academic progress" OR "knowledge acquisition" OR accomplishment OR attainment OR "academic success")) AND TITLE-ABS-KEY(("young adult*" OR students OR freshman OR "emerging adult*" OR college OR undergraduate))	365	91
**Cochrane Library**	(Mediterranean NEXT diet NEXT adherence OR Mediterranean NEXT scor* OR Mediterranean NEXT diet* OR MedDiet OR Mediterranean NEXT style NEXT diet* OR Mediterranean NEXT eat* OR Mediterranean NEXT food) AND (academic NEXT performance OR academic NEXT achievement OR learning OR educational OR intellectual OR knowledge OR excellence OR success OR academic NEXT progress OR knowledge NEXT acquisition OR accomplishment OR attainment OR academic NEXT success) AND (young NEXT adult OR students OR freshman OR emerging NEXT adult* OR college OR undergraduate*)	62	12
**Google Scholar** (search engine)	("young adult" OR student* OR freshman OR "emerging adult*" OR college OR undergraduate*) AND ("Mediterranean diet adherence" OR "Mediterranean scor*" OR "Mediterranean diet*" OR MedDiet OR "Mediterranean-style diet*" OR "Mediterranean eat*" OR "Mediterranean food") AND ("academic performance" OR "academic achievement" OR academic* OR "academic grades" OR "academic behaviour" OR "grade point average" OR "GPA" OR excellence OR success OR "academic progress" OR "knowledge acquisition" OR accomplishment OR attainment OR "academic success")	100 (first 10 pages screened)	200 (first 20 pages screened)

Notes. All sources (PubMed, Web of Science, Scopus, Cochrane Library, and Google Scholar) were initially searched on September 23, 2025, from inception, with no restrictions on publication date or language. The search was updated on August 6, 2026, and restricted to records published between September 23, 2025 and August 6, 2026, which explains the smaller number of records retrieved in the update. For Google Scholar, results were screened by pages, and the number of pages screened was increased in the update to ensure a more comprehensive search.

Table S4. Reason for exclusion after full-text screening (n = 83).

ID	Reference
**Reason for exclusion:** Wrong outcome	
**1**	Abasız, S.; Arslan, M. Adherence to Healthy Dietary Patterns and Cognitive Performance: Evidence from DASH and Mediterranean Diets. *Nutrients* **2026**, *18*, doi:10.3390/nu18111702.
**2**	Alfaro-González, S.; Garrido-Miguel, M.; Pascual-Morena, C.; Pozuelo-Carrascosa, D.P.; Fernández-Rodríguez, R.; Martínez-Hortelano, J.A.; Mesas, A.E.; Martínez-Vizcaíno, V. The Association Between Adherence to the Mediterranean Diet and Depression and Anxiety Symptoms in University Students: The Mediating Role of Lean Mass and the Muscle Strength Index. *Nutrients* **2025**, 17, doi:10.3390/nu17020346.
**3**	Chacón-Cuberos, R.; Zurita-Ortega, F.; Olmedo, E.M.; Padial-Ruz, R.; Castro-Sanchez, M. An Exploratory Model of Psychosocial Factors and Healthy Habits in University Students of Physical Education Depending on Gender. *International Journal of Environmental Research and Public Health* **2018**, 15, doi:10.3390/ijerph15112430.
**4**	Chacón-Cuberos, R.; Zurita-Ortega, F.; Olmedo, E.M.; Castro-Sanchez, M. Relationship between Academic Stress, Physical Activity and Diet in University Students of Education. *Behavioral Sciences* **2019**, 9, doi:10.3390/bs9060059.
**5**	Chacón-Cuberos, R.; Badicu, G.; Zurita-Ortega, F.; Castro-Sánchez, M. Mediterranean Diet and Motivation in Sport: A Comparative Study Between University Students from Spain and Romania. *Nutrients* **2018**, 11, doi:10.3390/nu11010030.
**6**	Delicado-Soria, A.; Serrano-Urrea, R.; Cervera-Burriel, F.; Daouas, T.; García-Meseguer, M.-J. Food Consumption in Tunisian University Students and Its Association with Sociodemographic Characteristics and Lifestyle Behaviours. *Public health nutrition* **2021**, 24, 4949–4964, doi:10.1017/S1368980020004942.
**7**	Ferreira-Pêgo, C.; Rodrigues, J.; Costa, A.; Sousa, B. Adherence to the Mediterranean Diet in Portuguese University Students. *Biomedical and Biopharmaceutical Research* **2019**, 16, 41–49, doi:10.19277/bbr.16.1.196.
**8**	Ferreira-Pêgo, C.; Rodrigues, J.; Costa, A.; Sousa, B. Eating Behavior: The Influence of Age, Nutrition Knowledge, and Mediterranean Diet. *Nutrition and Health* **2020**, 26, 303–309, doi:10.1177/0260106020945076.
**9**	Franchini, C.; Biasini, B.; Sogari, G.; Wongprawmas, R.; Andreani, G.; Dolgopolova, I.; Gómez, M.I.; Roosen, J.; Menozzi, D.; Mora, C.; et al. Adherence to the Mediterranean Diet and Its Association with Sustainable Dietary Behaviors, Sociodemographic Factors, and Lifestyle: A Cross-Sectional Study in US University Students. *Nutrition journal* **2024**, 23, 56, doi:10.1186/s12937-024-00962-0.

ID	Reference
**Reason for exclusion:** Wrong outcome	
10	García-Pérez, L.; Villodres, G.C.; Muros, J.J. Differences in Healthy Lifestyle Habits in University Students as a Function of Academic Area. *Journal of public health (Oxford, England)* **2023**, 45, 513–522, doi:10.1093/pubmed/fdac120.
11	González-Sosa, S.; Ruiz-Hernández, J.J.; Puente-Fernández, A.; Robaina-Bordón, J.M.; Conde-Martel, A. Adherence to the Mediterranean Diet in Medical Students. *Public Health Nutrition* **2023**, 26, 1798–1806, doi:10.1017/S1368980023000964.
12	Gürbüz, M.; Bayram, H.M.; Kabayel, N.; Türker, Z.S.; Şahin, Ş.; İçer, S. Association between Breakfast Consumption, Breakfast Quality, Mental Health and Quality of Life in Turkish Adolescents: A High School-Based Cross-Sectional Study. *Nutrition bulletin* **2024**, 49, 157–167, doi:10.1111/nbu.12668.
13	Hoyos Cillero, I.; Ruiz, I.L. Sociodemographic and Health Correlates of Health-Promoting Lifestyle Behaviors Among Nursing Students. *Nursing Reports* **2026**, *16*, doi:10.3390/nursrep16050150.
14	Ibáñez-del Valle, V.; Navarro-Martínez, R.; Cauli, O. Association between Depressive Symptoms and Adherence to the Mediterranean Diet in Nursing Students. *Nutrients* **2023**, 15, doi:10.3390/nu15143158.
15	Ilkay, H.O. Evening Chronotype, Irregular Circadian Eating Patterns, and Eating-Related Behaviors May Be Associated with Increased Obesity Risk among Turkish University Students: A Large-Scale Cross-Sectional Study. *Nutrition Research* **2025**, *144*, 74–87, doi:10.1016/j.nutres.2025.11.002.
16	Koutsonida, M.; Kanellopoulou, A.; Markozannes, G.; Gousia, S.; Doumas, M.T.; Sigounas, D.E.; Tzovaras, V.T.; Vakalis, K.; Tzoulaki, I.; Evangelou, E.; et al. Adherence to Mediterranean Diet and Cognitive Abilities in the Greek Cohort of Epirus Health Study. *Nutrients* **2021**, 13, doi:10.3390/nu13103363.
17	Kyprianidou, M.; Onoufriou, S.; Giannakou, K. Dietary Habits of Cypriot Students: A Cross-Sectional Study. *NUTRITION & FOOD SCIENCE* **2025**, *55*, 1169–1184, doi:10.1108/NFS-01-2025-0004.
18	Lo Moro, G.; Corezzi, M.; Bert, F.; Buda, A.; Gualano, M.R.; Siliquini, R. Mental Health and Adherence to Mediterranean Diet among University Students: An Italian Cross-Sectional Study. *Journal of American college health : J of ACH* **2023**, 71, 2451–2461, doi:10.1080/07448481.2021.1970567.
19	López-Olivares, M.; Sapp, P.; Riley, T.M.; Kris-Etherton, P.M.; Enrique-Mirón, C.; Nestares, T.; Davis, K.M. A Mediterranean Dietary Pattern Is Associated with Greater Participation in Physical Activity and Better Health-Related Quality of Life among Students and Professors at the Melilla Campus (University of Granada). *Nutrients* **2023**, 15, doi:10.3390/nu15183971.

ID	Reference
**Reason for exclusion:** Wrong outcome	
20	Marchena-Giráldez, M.; Bernabéu-Brotóns, B.; Iglesias-López, I.M. *Are Adherence to the Mediterranean Diet, Emotional Eating, Alcohol Intake, and Anxiety Related in University Students in Spain? Nutrients* **2020**, 12, 1–15, doi:10.3390/nu12082224.
21	Marconi, S.; Nucci, D.; Montani, G.; Gilberti, G.; Marullo, M.; Facciano, L.; Passeri, C.; Zanini, B. Improvement in Adherence to Mediterranean Diet, Cooking and Food Skills Among University Students Attending a "Teaching Kitchen" Project: Results from the S.A.P.O.R.E. Initiative. *FOODS* **2026**, *15*, doi:10.3390/foods15020302.
22	Martín-Espinosa, N.M.; Garrido-Miguel, M.; Martínez-Vizcaíno, V.; González-García, A.; Redondo-Tébar, A.; Cobo-Cuenca, A.I. The Mediating and Moderating Effects of Physical Fitness of the Relationship between Adherence to the Mediterranean Diet and Health-Related Quality of Life in University Students. *Nutrients* **2020**, 12, doi:10.3390/nu12113578.
23	Mata, S.S.R.; Zurita-Ortega, F.Z.; Martínez-Martínez, A.M.; Padial-Ruz, R.P.; Chacón-Cuberos, R.C.; Manrique, M.L. Adherence to Mediterranean Diet in University Students from Southern Spain Depending on Social and Academic Factors and Religious. *Revista Espanola de Nutricion Humana y Dietetica* **2018**, 22, 141–148, doi:10.14306/renhyd.22.2.446.
24	McEvoy, C.T.; Hoang, T.; Sidney, S.; Steffen, L.M.; Jacobs, D.R., Jr.; Shikany, J.M.; Wilkins, J.T.; Yaffe, K. Dietary Patterns during Adulthood and Cognitive Performance in Midlife: The CARDIA Study. *Neurology* **2019**, 92, e1589–e1599, doi:10.1212/WNL.0000000000007243.
25	Mieziene, B.; Emeljanovas, A.; Fatkulina, N.; Stukas, R. Dietary Pattern and Its Correlates among Lithuanian Young Adults: Mediterranean Diet Approach. *Nutrients* **2020**, 12, doi:10.3390/nu12072025.
26	Monserrat-Hernández, M.; Checa Olmos, J.C.; Arjona-Garrido, Á.; Lopez-Liria, R.; Rocamora-Pérez, P. Academic Stress in University Students: The Role of Physical Exercise and Nutrition. *Healthcare (Switzerland)* **2023**, 11, doi:10.3390/healthcare11172401.
27	Morales, G.; Balboa-Castillo, T.; Fernández-Rodríguez, R.; Garrido-Miguel, M.; Molino Guidoni, C.; Sirtoli, R.; Mesas, A.E.; Rodrigues, R. Adherence to the Mediterranean Diet and Depression, Anxiety, and Stress Symptoms in Chilean University Students: A Cross-Sectional Study. *Cadernos de saude publica* **2023**, 39, e00206722, doi:10.1590/0102-311XEN206722.
28	Moustafa, B.; Trifan, G.; Isasi, C.R.; Lipton, R.B.; Sotres-Alvarez, D.; Cai, J.; Tarraf, W.; Stickel, A.; Mattei, J.; Talavera, G.A.; et al. Association of Mediterranean Diet With Cognitive Decline Among Diverse Hispanic or Latino Adults From the Hispanic Community Health Study/Study of Latinos. *JAMA network open* **2022**, 5, e2221982, doi:10.1001/jamanetworkopen.2022.21982.
29	Pennisi, F.; Nucci, D.; Pinto, A.; Stacchini, L.; Veronese, N.; Maggi, S.; Signorelli, C.; Baldo, V.; Gianfredi, V. Adherence to the Mediterranean Diet and Depressive Symptoms. A Cross-Sectional Study among Italian University Students: The UniFoodWaste Study. *Nutrition* **2026**, *144*, 113070, doi:10.1016/j.nut.2025.113070.

ID	Reference
**Reason for exclusion:** Wrong outcome	
**30**	Pérez-Gallardo, L.; Mingo-Gómez, T.M.; Bayona-Marzo, I.B.; Pascual, M.Á.F.; Márquez, E.M.; Domínguez, R.R.; Navas-Ferrer, C.; Cámara, F.N. Diet Quality in College Students with Different Academic Profile. *Nutricion Hospitalaria* **2015**, 31, 2230–2239, doi:10.3305/nh.2015.31.5.8614.
**31**	Pérez-Mármol, M.; Chacón-Cuberos, R.; García-Mármol, E.; Castro-Sánchez, M. Relationships among Physical Self-Concept, Physical Activity and Mediterranean Diet in Adolescents from the Province of Granada. *Children (Basel, Switzerland)* **2021**, 8, doi:10.3390/children8100901.
**32**	Pérez-Mármol, M.; Castro-Sanchez, M.; Chacón-Cuberos, R.; Gamarra-Vengoechea, M.A. Relationship between Academic Performance, Psychosocial Factors and Healthy Habits in Secondary School Students. *Aula Abierta* **2023**, 52, 281–288, doi:10.17811/rifie.52.3.2023.281-288.
**33**	Quarta, S.; Siculella, L.; Levante, A.; Carluccio, M.A.; Calabriso, N.; Scoditti, E.; Damiano, F.; Lecciso, F.; Pinto, P.; García-Conesa, M.-T.; et al. Association between Mediterranean Lifestyle and Perception of Well-Being and Distress in a Sample Population of University Italian Students. *International journal of food sciences and nutrition* **2023**, 74, 556–567, doi:10.1080/09637486.2023.2232129.
**34**	Restović, I.; Jukić, A.; Kević, N. The Mediterranean Paradox: Knowledge, Attitudes, and the Barriers to Practical Adherence of Sustainable Dietary Behavior Among Future Educators—A Case Study of Teacher Education Students at the University of Split. *Sustainability (Switzerland)* **2026**, *18*, doi:10.3390/su18020831.
**35**	Sanchis, C.; Plaza, M.; Checa, I.; Monleón, C. Combined Effects of a Mediterranean Diet and Respiratory Muscle Training on Higher Education Woodwind Musicians: A Randomized Controlled Trial. *Heliyon* **2024**, 10, e35495, doi:10.1016/j.heliyon.2024.e35495.
**36**	Sicinska, E.; Madej, D.; Szmidt, M.K.; Januszko, O.; Kaluza, J. Dietary Supplement Use in Relation to Socio-Demographic and Lifestyle Factors, Including Adherence to Mediterranean-Style Diet in University Students. *Nutrients* **2022**, 14, doi:10.3390/nu14132745.
**37**	Tárraga López, P.J.; Tárraga Marcos, A.; Panisello, J.M.; Herencia Carbayo, J.A.; Tárraga Marcos, M.L.; López-Gil, J.F. Physical Activity and Its Association with Mediterranean Diet Patterns among Spanish University Students. *Nutricion hospitalaria* **2022**, 39, 603–609, doi:10.20960/nh.03892.
**38**	Tomás-Gallego, G.; Dalmau-Torres, J.M.; Jiménez-Boraita, R.; Ortuño-Sierra, J.; Gargallo-Ibort, E. Adherence to the Mediterranean Diet in Spanish University Students: Association with Lifestyle Habits, Mental and Emotional Well-Being. *Nutrients* **2025**, 17, doi:10.3390/nu17040698.
**39**	Tomás-Gallego, G.; Jiménez Boraita, R.; Ortuño-Sierra, J.; Gargallo Ibort, E.; Dalmau Torres, J.M. Emotional and Behavioural Problems in Spanish University Students: Association with Lifestyle Habits and Mental Well-Being. *Healthcare (Switzerland)* **2024**, 12, doi:10.3390/healthcare12151482.

ID	Reference
**Reason for exclusion:** Wrong outcome	
**40**	Trigueros, R.; Padilla, A.M.; Aguilar-Parra, J.M.; Rocamora-Pérez, P.; Morales-Gázquez, M.J.; Lopez-Liria, R. The Influence of Emotional Intelligence on Resilience, Test Anxiety, Academic Stress and the Mediterranean Diet. A Study with University Students. *International Journal of Environmental Research and Public Health* **2020**, 17, doi:10.3390/ijerph17062071.
**41**	Ubago-Jiménez, J.L.; Zurita-Ortega, F.; San Román-Mata, S.; Puertas-Molero, P.; González-Valero, G. Impact of Physical Activity Practice and Adherence to the Mediterranean Diet in Relation to Multiple Intelligences among University Students. *Nutrients* **2020**, 12, doi:10.3390/nu12092630.
**42**	Ugartemendia, L.; Bravo, R.; Castaño, M.Y.; Cubero, J.; Zamoscik, V.; Kirsch, P.; Rodríguez, A.B.; Reuter, M. "Influence of Diet on Mood and Social Cognition: A Pilot Study". *Food & function* **2020**, 11, 8320–8330, doi:10.1039/d0fo00620c.
**43**	Zaitoon, H.; Kaly, L.; Khalil, H.; Zion, N. Balancing Knowledge and Health: A Comparative Analysis of Students and Healthcare Workers Nutrition Related Health Behaviors, a Cross-Sectional Study. *Nursing and Health Sciences* **2024**, 26, doi:10.1111/nhs.70000.
**Reason for exclusion:** Wrong population	
**44**	Alonso-Vargas, J.M.A.; González-Valero, G.G.; Puertas, P.P.; Salvador-Pérez, F.S.; Melguizo-Ibáñez, E.M. Relationship between Learning and Healthy Habits in Early Childhood Education. *Retos* **2023**, 48, 161–166, doi:10.47197/retos.v48.96658.
**45**	Arab, L.; Ang, A. A Cross Sectional Study of the Association between Walnut Consumption and Cognitive Function among Adult Us Populations Represented in NHANES. *Journal of Nutrition, Health and Aging* **2015**, 19, 284–290, doi:10.1007/s12603-014-0569-2.
**46**	Brouwer-Brolsma, E.M.; Benati, A.; van de Wiel, A.; van Lee, L.; de Vries, J.H.M.; Feskens, E.J.M.; van de Rest, O. Higher Mediterranean Diet Scores Are Not Cross-Sectionally Associated with Better Cognitive Scores in 20- to 70-Year-Old Dutch Adults: The NQplus Study. *Nutrition research (New York, N.Y.)* **2018**, 59, 80–89, doi:10.1016/j.nutres.2018.07.013.
**47**	Cano-Cañada, E.; Llanos-Muñoz, R.; Rubio-Morales, A.; Lobo-Triviño, D.; Tapia-Serrano, M.Á. Influence of Healthy Lifestyles on Academic Engagement and Academic Performance in Adolescents. *Revista Andaluza de Medicina del Deporte* **2023**, 16, 118–124, doi:10.33155/ramd.v16i3-4.1026.

Table S4. *Cont.*

ID	Reference
**Reason for exclusion:** Wrong population	
**48**	Enriquez-del Castillo, L.; Villegas-Balderrama, C.; López-Alonzo, S.; Flores, L.; Martinez-Trevizo, A.; Islas-Guerra, S. Planetary Health Diet versus Usual Diet in Adolescents. *How Do Food and Physical Activity Influence Academic Performance? NUTRICION HOSPITALARIA* **2024**, 41, 28–37, doi:10.20960/nh.04614.
**49**	Gardener, S.L.; Rainey-Smith, S.R.; Barnes, M.B.; Sohrabi, H.R.; Weinborn, M.; Lim, Y.Y.; Harrington, K.; Taddei, K.; Gu, Y.; Rembach, A.; et al. Dietary Patterns and Cognitive Decline in an Australian Study of Ageing. *Molecular psychiatry* **2015**, 20, 860–866, doi:10.1038/mp.2014.79.
**50**	Gaynor, A.M.; Varangis, E.; Song, S.; Gazes, Y.; Noofoory, D.; Babukutty, R.S.; Habeck, C.; Stern, Y.; Gu, Y. Diet Moderates the Effect of Resting State Functional Connectivity on Cognitive Function. *Scientific reports* **2022**, 12, 16080, doi:10.1038/s41598-022-20047-4.
**51**	Hayek, J.; de Vries, H.; Tueni, M.; Lahoud, N.; Winkens, B.; Schneider, F. Increased Adherence to the Mediterranean Diet and Higher Efficacy Beliefs Are Associated with Better Academic Achievement: A Longitudinal Study of High School Adolescents in Lebanon. *INTERNATIONAL JOURNAL OF ENVIRONMENTAL RESEARCH AND PUBLIC HEALTH* **2021**, 18, doi:10.3390/ijerph18136928.
**52**	Hayek, J.; Schneider, F.; Tueni, M.; de Vries, H. Is Academic Achievement Related to Mediterranean Diet, Substance Use and Social-Cognitive Factors: Findings from Lebanese Adolescents. *NUTRIENTS* **2020**, 12, doi:10.3390/nu12051535.
**53**	Hrezova, E.; Stefler, D.; Capkova, N.; Vaclova, H.; Bobak, M.; Pikhart, H. Mediterranean Diet Score Linked to Cognitive Functioning in Czech Women: A Cross-Sectional Population-Based Study. *European journal of nutrition* **2025**, 64, 237, doi:10.1007/s00394-025-03752-4.
**54**	Jiménez Boraita, R.; Arriscado Alsina, D.; Dalmau Torres, J.M.; Gargallo Ibort, E. Factors Associated with Night Sleep in Adolescents. *Pediatria de Atencion Primaria* **2022**, 24, 375–384.
**55**	Jiménez Boraita, R.; Dalmau Torres, J.M.; Gargallo Ibort, E.; Arriscado Alsina, D. Factors Associated with Academic Performance in Adolescents from La Rioja (Spain): Lifestyle Habits, Health Indicators, and Sociodemographic Factors. *Nutrición Hospitalaria* **2024**, 41, 19–27, doi:10.20960/nh.04599.
**56**	Jiménez Boraita, R.; Gargallo Ibort, E.; Dalmau Torres, J.M.; Arriscado Alsina, D. Factors Associated with a Low Level of Physical Activity in Adolescents from La Rioja (Spain). *Anales de Pediatria* **2022**, 96, 326–333, doi:10.1016/j.anpedi.2021.02.011.

Table S4. *Cont.*

ID	Reference
**Reason for exclusion:** Wrong population	
**57**	Jiménez Boraita, R.J.; Arriscado Alsina, D.A.; Gargallo Ibort, E.G.; Dalmau Torres, J.M.D. Determinants of Health in Adolescence: Cardiorespiratory Fitness and Body Composition. *Nutricion Hospitalaria* **2021**, 38, 697–703, doi:10.20960/nh.03507.
**58**	Jiménez Boraita, R.J.; Arriscado Alsina, D.A.; Torres, J.M.D.; Gargallo Ibort, E.G. Determinants of Body Satisfaction in Adolescents. *Revista Española de Salud Pública* **2021**, 95.
**59**	Jiménez Boraita, R.J.; Gargallo Ibort, E.G.; Arriscado Alsina, D.A.; Torres, J.M.D. Association between the Physical Activity Practice Environment and Lifestyle Habits and Indicators of Physical and Psychosocial Health. *Revista Española de Salud Pública* **2022**, 96.
**60**	Jiménez Boraita, R.; Gargallo Ibort, E.; Dalmau Torres, J.M.; Arriscado Alsina, D. Lifestyle Habits, Health Indicators and Sociodemographic Factors Associated with Health-Related Quality of Life and Self-Esteem in Adolescents. *Clinical child psychology and psychiatry* **2024**, 29, 493–512, doi:10.1177/13591045231200661.
**61**	Jiménez-López, E.; Mesas, A.E.; Visier-Alfonso, M.E.; Pascual-Morena, C.; Martínez-Vizcaíno, V.; Herrera-Gutiérrez, E.; López-Gil, J.F. Adherence to the Mediterranean Diet and Depressive, Anxiety, and Stress Symptoms in Spanish Adolescents: Results from the EHDLA Study. *European child & adolescent psychiatry* **2024**, 33, 2637–2646, doi:10.1007/s00787-023-02351-0.
**62**	Mukhamedzhanov, E.; Tsitsurin, V.; Zhakiyanova, Z.; Altynsarin, B.; Tarjibayeva, S. The Effect of Nutrition Education on Nutritional Behavior, Academic and Sports Achievement and Attitudes. *INTERNATIONAL JOURNAL OF EDUCATION IN MATHEMATICS SCIENCE AND TECHNOLOGY* **2023**, 11, 358–374, doi:10.46328/ijemst.3133.
**63**	Onetti-Onetti, W.; Álvarez-Kurogi, L.; Castillo, A. Adherence to the Mediterranean Diet Pattern and Self-Concept in Adolescents. *Nutricion Hospitalaria* **2019**, 36, 658–664, doi:10.20960/nh.2214.
**64**	Pérez-Mármol, M.; Castro-Sanchez, M.; Gamarra-Vengoechea, M.A.; Chacón-Cuberos, R. Are Self-Concept and Emotional Intelligence Associated with Healthy Habits and School Performance? A Structural Model According to Sex. *Revista de Investigacion Educativa* **2025**, 43, doi:10.6018/rie.590051.
**65**	Pérez-Mármol, M.; Chacón-Cuberos, R.; Belmonte-Arévalo, A.B.; Gamarra-Vengoechea, M.A.; Castro-Sanchez, M. ACADEMIC PERFORMANCE AND SELF-CONCEPT AS MEDIATORS OF HEALTHY HABITS: LINEAR MODEL IN ADOLESCENTS. *Journal of Sport and Health Research* **2024**, 16, 127–138, doi:10.58727/jshr.98353.

Table S4. *Cont.*

ID	Reference
**Reason for exclusion:** Wrong population	
66	Qin, Y.; Lu, M.; Liu, L.; Li, M.; Mi, B.; Zhou, J.; Xiao, Y. The Effects of Diet Quality, Eating Behavior, and Sleep on the Academic Performance among Elementary School Students. *ECOLOGY OF FOOD AND NUTRITION* **2024**, 63, 472–488, doi:10.1080/03670244.2024.2371154.
67	Rosi, A.; Giopp, F.; Milioli, G.; Melegari, G.; Goldoni, M.; Parrino, L.; Scazzina, F. Weight Status, Adherence to the Mediterranean Diet, Physical Activity Level, and Sleep Behavior of Italian Junior High School Adolescents. *Nutrients* **2020**, 12, doi:10.3390/nu12020478.
68	Vassiloudis, I.; Yiannakouris, N.; Panagiotakos, D.B.; Apostolopoulos, K.; Costarelli, V. Academic Performance in Relation to Adherence to the Mediterranean Diet and Energy Balance Behaviors in Greek Primary Schoolchildren. *Journal of nutrition education and behavior* **2014**, 46, 164–170, doi:10.1016/j.jneb.2013.11.001.
69	Vassiloudis, I.; Yiannakouris, N.; Panagiotakos, D.B.; Apostolopoulos, K.; Costarelli, V. Adherence to the Mediterranean Diet and Specific Lifestyle Habits Are Associated with Academic Performance in Greek Adolescents. *Mediterranean Journal of Nutrition and Metabolism* **2017**, 10, 93–103, doi:10.3233/MNM-16133.
70	Villodres, G.; Pérez-Díaz, J.; Salas-Montoro, J.; Muros, J. Sex-Based Differences in Lifestyle Behaviours, Self-Esteem, and Academic Performance: A Structural Equation Model in High-Socioeconomic-Status School-Aged Youth from Southern Spain. *CHILDREN-BASEL* **2025**, *12*, doi:10.3390/children12111459.
**Reason for exclusion:** Wrong exposure	
71	Belogianni, K.; Ooms, A.; Lykou, A.; Moir, H. Nutrition Knowledge among University Students in the UK: A Cross-Sectional Study. *PUBLIC HEALTH NUTRITION* **2022**, 25, 2834–2841, doi:10.1017/S1368980021004754.
72	de Mier, G.; Estevan, M.; Magdalena, C.; de Diego, J.; Herreros, P. University Students' Food Consumption Assessment and the Relation with Their Academic Profile. *NUTRICION HOSPITALARIA* **2017**, 34, 134–143, doi:10.20960/nh.989.
73	Guiné, R.; Florença, S.; Aparício, M.; Cardoso, A.; Ferreira, M. Food Knowledge for Better Nutrition and Health: A Study among University Students in Portugal. *HEALTHCARE* **2023**, 11, doi:10.3390/healthcare11111597.
74	López-Moreno, M.; Garcés-Rimón, M.; Miguel, M.; Iglesias-López, M. Influence of Eating Habits and Alcohol Consumption on the Academic Performance among a University Population in the Community of Madrid: A Pilot Study. *HELIYON* **2021**, 7, doi:10.1016/j.heliyon.2021.e07186.

Table S4. *Cont.*

ID	Reference
**Reason for exclusion:** Ineligible study association	
75	Al-Amouri, F.M.; Qurom, S.; Daoud, M.; Daas, M.; Shbaitah, Y.; Badrasawi, M. Prevalence and Associated Factors of Attention Deficit Hyperactivity Disorder Symptoms among Palestinian University Students. *Journal of public health research* **2025**, 14, 22799036251373010, doi:10.1177/22799036251373010.
76	Alexatou, O.; Papadimitriou, K.; Angelakou, E.; Papadopoulou, S.; Pappa, M.; Ntovoli, A.; Serdari, A.; Apostolidou, K.; Vorvolakos, T.; Giaginis, C. The Relationship Between Obesity, Overweight, and the Prevalence of Depression and Anxiety Among University Students: Evidence from a Nationally Representative Cross-Sectional Study in Greece. *DISEASES* **2026**, *14*, doi:10.3390/diseases14040136.
77	Alotaibi, M.I.; Elsamad, G.; Aljardahi, A.N.; Alghamdi, A.N.; Alotaibi, A.I.; Alorabi, H.M.; Alzahrani, K.M.; Abdel-Moneim, A.S. Changes in Dietary and Lifestyle Behaviors and Mental Stress among Medical Students upon Ramadan Diurnal Intermittent Fasting: A Prospective Cohort Study from Taif/Saudi Arabia. *BMC Public Health* **2023**, 23, doi:10.1186/s12889-023-16385-1.
78	Benavides-Sánchez, M.J.; Ubago Jiménez, J.L.; Melguizo-Ibáñez, E.; Alonso-Vargas, J.M. Influence of Physical Activity and Diet on Stress and Anxiety in Higher Education. *Sport TK* **2024**, 13, doi:10.6018/sportk.636041.
79	Cena, H.; Porri, D.; De Giuseppe, R.; Kalmpourtzidou, A.; Salvatore, F.P.; El Ghoch, M.; Itani, L.; Kreidieh, D.; Brytek-Matera, A.; Pocol, C.B.; et al. How Healthy Are Health-Related Behaviors in University Students: The HOLISTic Study. *Nutrients* **2021**, 13, doi:10.3390/nu13020675.
**Reason for exclusion:** Duplicate results of interest	
80	Alexatou, O.; Voulgaridou, G.; Papadopoulou, S.K.; Jacovides, C.; Serdari, A.; Deligiannidou, G.-E.; Tsourouflis, G.; Pappa, M.; Vorvolakos, T.; Giaginis, C. Mediterranean Diet Adherence Is Associated with Lower Prevalence of Depression and Anxiety in University Students: A Cross-Sectional Study in Greece. *Diseases* **2026**, *14*, 19, doi:10.3390/diseases14010019.
81	Melguizo-Ibáñez, E.; González-Valero, G.; Badicu, G.; Yagin, F.H.; Alonso-Vargas, J.M.; Ardigò, L.P.; Puertas-Molero, P. Mediterranean Diet Adherence on Self-Concept and Anxiety as a Function of Weekly Physical Activity: An Explanatory Model in Higher Education. *Frontiers in nutrition* **2023**, *10*, 1215359, doi:10.3389/fnut.2023.1215359.
**Reason for exclusion:** Inconsistent results	
82	Eroğlu, H.A.; Aykora, D.; Sert, B.; Aydeğer, C. Sleep, Diet, and Academic Success: A Cross-Sectional Study Among Medical Students. *Kafkas J Med Sci* **2025**, *15*, 282–287, doi:10.5505/kjms.2025.63004.
83	Fernández-Medina, I.M.; Ruíz-Fernández, M.D.; Hernández-Padilla, J.M.; Granero-Molina, J.; Fernández-Sola, C.; Jiménez-Lasserrotte, M.d.M.; Lirola, M.-J.; Cortés-Rodríguez, A.E.; López-Rodríguez, M.M. Adherence to the Mediterranean Diet and Self-Efficacy as Mediators in the Mediation of Sleep Quality and Grades in Nursing Students. *Nutrients* **2020**, *12*, doi:10.3390/nu12113265.

Table S5. Main findings, effect estimates, and Mediterranean diet adherence distribution of included studies.

<table>
<tr><th>Study (year)</th><th>MedDiet adherence distribution</th><th>AP outcome (type)</th><th>Raw data extracted</th><th>Computed statistic</th><th>Result</th><th>Direction of association</th><th>Adjustment set</th></tr>
<tr><td>Alfaro-González et al., 2024 [3]</td><td>Low: 76.4% (n = 203)
Good: 23.6% (n = 63)
Mean MEDAS: 7.05 ± 2.06</td><td>Objective</td><td>Fully adjusted means ± SD
Low (MEDAS <P25): -0.24 ± 1.02
High (MEDAS >P75): 0.32 ± 1.15</td><td>Cohen's d (95% CI)</td><td>0.52 (0.17; 0.86)</td><td>Positive, significant</td><td>Age, sex, location, tobacco consumption, total energy intake, BMI, cardiorespiratory fitness, MVPA, maternal education level, type of housing</td></tr>
<tr><td>Dakanalis et al., 2025 [4]</td><td>Poor: 47.6% (n = 2,589)
Average: 34.5% (n = 1,873)
Good: 17.9% (n = 971)</td><td>Objective</td><td>Category frequencies (2×2)<table><tr><th>n</th><th>Lower AP</th><th>Higher AP</th></tr><tr><td>Lower MedDiet</td><td>1,545</td><td>203</td></tr><tr><td>Higher MedDiet</td><td>249</td><td>541</td></tr></table></td><td>OR (95% CI)</td><td>16.54 (13.41; 20.39)</td><td>Positive, significant</td><td>None</td></tr>
<tr><td>El Hajj and Julien, 2021 [5]</td><td>Poor: 32.7% (n = 99)
Average: 48.5% (n = 147)
High: 18.8% (n = 57)</td><td>Objective</td><td>Category frequencies (2×2)<table><tr><th>n</th><th>Lower AP</th><th>Higher AP</th></tr><tr><td>Lower MedDiet</td><td>4</td><td>17</td></tr><tr><td>Higher MedDiet</td><td>1</td><td>24</td></tr></table></td><td>OR (95% CI)</td><td>5.65 (0.58; 55.08)</td><td>Positive, non-significant</td><td>None</td></tr>
<tr><td>Esin and Ayyıldız, 2024 [6]</td><td>Mean MEDAS score: 6.0 ± 2.02 (food secure); 5.3 ± 1.82 (food insecure); overall distribution NR</td><td>Objective</td><td>Correlation coefficient</td><td>Pearson's r (p-value)</td><td>0.018 (>0.05)</td><td>Positive, non-significant</td><td>None</td></tr>
<tr><td>Gianfredi et al., 2018 [7]</td><td>Low: 47.0% (n = 55)
Moderate: 48.7% (n = 57)
High: 4.3% (n = 5)
Mean MEDAS: 5.9 ± 2.1</td><td>Subjective</td><td>Correlation coefficient</td><td>Pearson's r (p-value)</td><td>0.247 (0.009)</td><td>Positive, significant</td><td>None</td></tr>
<tr><td>Gimeno Tena and Esteve Clavero, 2021 [8]</td><td>Low: 39.5% (n = 45)
Medium: 60.5% (n = 69)
High: 0% (n = 0)
Mean MEDAS: 6.0 ± 1.71</td><td>Objective</td><td>Category frequencies (2×2)<table><tr><th>n</th><th>Lower AP</th><th>Higher AP</th></tr><tr><td>Lower MedDiet</td><td>24</td><td>21</td></tr><tr><td>Higher MedDiet</td><td>29</td><td>40</td></tr></table></td><td>OR (95% CI)</td><td>1.58 (0.74; 3.37)</td><td>Positive, non-significant</td><td>None</td></tr>
</table>

Continued on next page …

Table S5. *Cont.*

<table>
<tr><th>Study (year)</th><th>MedDiet adherence distribution</th><th>AP outcome (type)</th><th>Raw data extracted</th><th>Computed statistic</th><th>Result</th><th>Direction of association</th><th>Adjustment set</th></tr>
<tr><td>López-Nuevo et al., 2021 [9]</td><td>Distribution by category NR;
KIDMED score reported as median by subgroup:
APSA (n = 28): 8.00 (IQR 7.00-10.00)
DPPA (n = 27): 6.00 (IQR 4.00–8.00)</td><td>Objective</td><td>Correlation coefficient</td><td>Spearman’s ρ (p-value)</td><td>0.280 (0.100)</td><td>Non-significant in the original study; significant as a converted effect size in the meta-analysis [a]</td><td>None</td></tr>
<tr><td>Melguizo-Ibáñez et al., 2022 [10]</td><td>Low: 3.4% (n = 19)
Medium: 56.8% (n = 317)
Optimum: 39.8% (n = 222)</td><td>(i) Objective
(ii) Subjective</td><td>(i) Correlation coefficient
(ii) Means ± SD
Low (MEDAS ≤7): 3.86 ± 0.63
Optimal (MEDAS >10): 4.06 ± 0.66</td><td>(i) Pearson’s r (p-value)
(ii) Cohen’s d (95% CI)</td><td>(i) 0.106 (0.005)
(ii) 0.30 (-0.16; 0.77)</td><td>Positive, mixed (objective AP significant; subjective AP non-significant)</td><td>None</td></tr>
<tr><td>Naja et al., 2022 [11]</td><td>Distribution by individual category NR
Low: 45.9% (n = 231)
Medium + High: 54.1% (n = 272)</td><td>Objective</td><td>Category frequencies (2×2)<table><tr><th>n</th><th>Lower AP</th><th>Higher AP</th></tr><tr><td>Lower MedDiet</td><td>103</td><td>128</td></tr><tr><td>Higher MedDiet</td><td>116</td><td>156</td></tr></table></td><td>OR (95% CI)</td><td>1.08 (0.76; 1.54)</td><td>Positive, non-significant</td><td>None</td></tr>
<tr><td>Ünal et al., 2017 [12]</td><td>Poor: 37.5% (n = 137)
Average: 44.7% (n = 163)
Good: 17.8% (n = 65)</td><td>Objective</td><td>Means ± SD
Low (KIDMED ≤3): 2.72 ± 0.48
Optimal (KIDMED ≥8): 3.13 ± 0.41</td><td>Cohen’s d (95% CI)</td><td>0.89 (0.59; 1.20)</td><td>Positive, significant</td><td>None</td></tr>
<tr><td>Vujačić et al., 2025 [13]</td><td>Distribution by individual category NR
Mean KIDMED score: 4.70 overall (Montenegro: 4.98; Croatia: 4.75; Slovenia: 4.38)</td><td>Objective</td><td>Correlation coefficient</td><td>Pearson's r (p-value)</td><td>0.039 (0.348)</td><td>Positive, non-significant</td><td>None</td></tr>
<tr><td>Zurita-Ortega et al., 2018 [14]</td><td>Low: 0.5% (n = 3);
Medium: 21.9% (n = 131)
High: 77.6% (n = 463)</td><td>Subjective</td><td>Means ± SD
Low (KIDMED ≤1): 3.50 ± 1.25
High (KIDMED ≥8): 3.67 ± 0.58</td><td>Cohen’s d (95% CI)</td><td>0.29 (−0.84; 1.43)</td><td>Positive, non-significant</td><td>None</td></tr>
</table>

Notes: Values are shown as reported in the original studies unless otherwise indicated; correlation coefficients are shown with their *p*-value and all other estimates with their 95% CI. The direction of association reflects the relationship between higher MedDiet adherence and better AP (positive, inverse, or null), with significance set at $p < 0.05$. Estimates not reported in the original studies were derived by the review authors from the extracted raw data (odds ratios from 2×2 category frequencies; Cohen's *d* from group means and SDs) and then converted to Cohen's *d* for pooling. The "Adjustment set" column lists the covariates reflected in the estimate used in this meta-analysis, not the full adjustment of the original study. ***Abbreviations:*** AP, academic performance; APSA, Animation and Physical-Sports Activities cycle; BMI, body mass index; CI, confidence interval; DPPA, Dental Pathology and Prosthetic Anatomy cycle; IQR, interquartile range; KIDMED, Mediterranean Diet Quality Index in Children and Adolescents; MedDiet, Mediterranean diet; MEDAS, Mediterranean Diet Adherence Screener; MVPA, moderate-to-vigorous physical activity; *n*, sample size; NR, not reported; OR, odds ratio; *p*, probability value; SD, standard deviation. [a] Reported as Spearman's ρ (ρ = 0.280, *p*-value = 0.100) and treated as a correlation coefficient for conversion to Cohen's d. The converted effect size used in the meta-analysis was significant (SMD = 0.58, 95% CI 0.02–1.15), which may differ from the significance of the original correlation.

Table S6. Quality assessment of included studies [15].

Study (year)	1	2	3	4	5	6	7	8	9	10	11	12	13	14	Quality
Alfaro-González et al., 2024 [3]	Y	Y	Y	Y	N	NA	NA	Y	Y	N	Y	NR	NA	Y	Good
Dakanalis et al., 2025 [4]	Y	Y	Y	Y	Y	NA	NA	Y	Y	N	Y	NR	NA	N*	Good
El Hajj and Julien, 2021 [5]	Y	Y	CD	Y	CD	NA	NA	Y	Y	N	Y	NR	NA	N	Fair
Esin and Ayyıldız, 2024 [6]	Y	Y	CD	Y	Y	NA	NA	Y	Y	N	Y	NR	NA	N*	Fair
Gianfredi et al., 2018 [7]	Y	Y	Y	Y	N	NA	NA	Y	Y	N	N	NR	NA	N*	Fair
Gimeno Tena and Esteve Clavero, 2021 [8]	Y	Y	NR	Y	Y	NA	NA	CD	Y	N	Y	NR	NA	N	Fair
López-Nuevo et al., 2021 [9]	Y	Y	Y	Y	N	NA	NA	Y	Y	N	Y	NR	NA	N	Fair
Melguizo-Ibáñez et al., 2022 [10]	Y	Y	CD	Y	Y	NA	NA	Y	Y	N	Y	NR	NA	N	Fair
Naja et al., 2022 [11]	Y	Y	CD	Y	Y	NA	NA	Y	Y	N	Y	NR	NA	N*	Fair
Ünal et al., 2017 [12]	Y	Y	NR	Y	N	NA	NA	Y	Y	N	Y	NR	NA	N	Fair
Vujačić et al., 2025 [13]	Y	Y	NR	Y	N	NA	NA	Y	Y	N	Y	NR	NA	N	Fair
Zurita-Ortega et al., 2018 [14]	Y	Y	Y	Y	N	NA	NA	Y	Y	N	Y	NR	NA	N	Fair

Notes: Numbers represent the questions included in the National Institutes of Health's Quality Assessment Tool for Observational Cohort and Cross-Sectional Studies: 1. Question clear? 2. Population clearly defined? 3. >50% participation rate? 4. Recruitment populations consistent? 5. Sample size justified? 6. Exposure assessed prior to outcome? 7. Sufficient timeframe? 8. Different exposure levels? 9. Valid exposure? 10. Repeated exposure assessment? 11. Valid outcomes? 12. Outcome assessors blinded? 13. Loss to follow up; 14. Confounders adjusted for? Items 6, 7 and 13 was considered not applicable for cross-sectional studies. Each cross-sectional and longitudinal study was rated as good, fair, or poor according to the National Institutes of Health's Quality Assessment Tool for Observational Cohort and Cross-Sectional quality rating guide. * Although these studies reported covariate-adjusted analyses, the estimate used in the present review for the MedDiet–AP association was unadjusted, because the adjusted models addressed other associations or modeled AP as the predictor of MedDiet adherence. Item 14 was therefore rated N to reflect the absence of adjustment for the association of interest, regardless of the overall analytical approach of these studies. ***Abbreviations:*** CD, cannot determine; N, no; NA, not applicable; NR, not reported; Y, yes.

Table S7. Quality of evidence assessment according to the GRADE approach [16].

Outcome	Certainty assessment							No. of patients	Effect size (95% CI)	Certainty	Importance
	No. of studies	Study design	Risk of bias	Inconsistency	Indirectness	Imprecision	Other considerations				
Objective AP	10	non-randomised studies cross-sectional	serious[a]	very serious[17],[b]	very serious[c]	serious[d]	none	10,217	SMD **0.49 SD higher** (0.04 higher to 0.94 higher)[18],[e]	⨁◯◯◯ Very low[17],[a,b,c,d]	CRITICAL

Abbreviations: AP, academic performance; CI, confidence interval; GPA, grade point average; KIDMED, Mediterranean Diet Quality Index in Children and Adolescents; MEDAS, Mediterranean Diet Adherence Screener; SD, standard deviation; SMD, standardized mean difference.

Explanations

a. Downgraded by 1 level for serious risk of bias. Based on the NIH Quality Assessment Tool for Observational Cohort and Cross-Sectional Studies, most included studies were rated as fair quality (8/10), with 2 rated as good. Key limitations were consistent across studies: all used cross-sectional designs precluding causal inference; dietary adherence relied exclusively on self-reported instruments; outcome assessors were not blinded, and most studies failed to adequately adjust for potential confounders.

b. Downgraded by 2 levels for very serious inconsistency. The pooled analysis revealed considerable heterogeneity across studies ($I^2 = 98.2\%$, p-value < 0.001), as defined by the Cochrane Handbook for Systematic Reviews of Interventions [17].

c. Downgraded by 2 levels for very serious indirectness. Mediterranean diet adherence was assessed using two instruments with different item composition and scoring ranges (KIDMED and MEDAS-14). Academic performance was measured heterogeneously across studies, including GPA, mean academic grades, and university entrance examination scores on different scales. Moreover, in several studies this association was explored as a secondary aim rather than the primary research question.

d. Downgraded by 1 level for serious imprecision. The 95% CI of the pooled effect estimate (SMD 0.49; 95% CI 0.04 to 0.94) marginally excludes the null and spans a range from trivially small to large effect magnitudes, with the lower bound (SMD 0.04). The prediction interval (-1.17 to 2.15) crosses zero in both directions.

e. Effect expressed as a pooled standardized mean difference (SMD) from a random-effects meta-analysis (10 studies; $N = 10{,}217$). An SMD of 0.49 corresponds to a small-to-moderate effect size per Cohen's conventions [18]. A positive SMD indicates higher Mediterranean diet adherence associated with better academic performance.

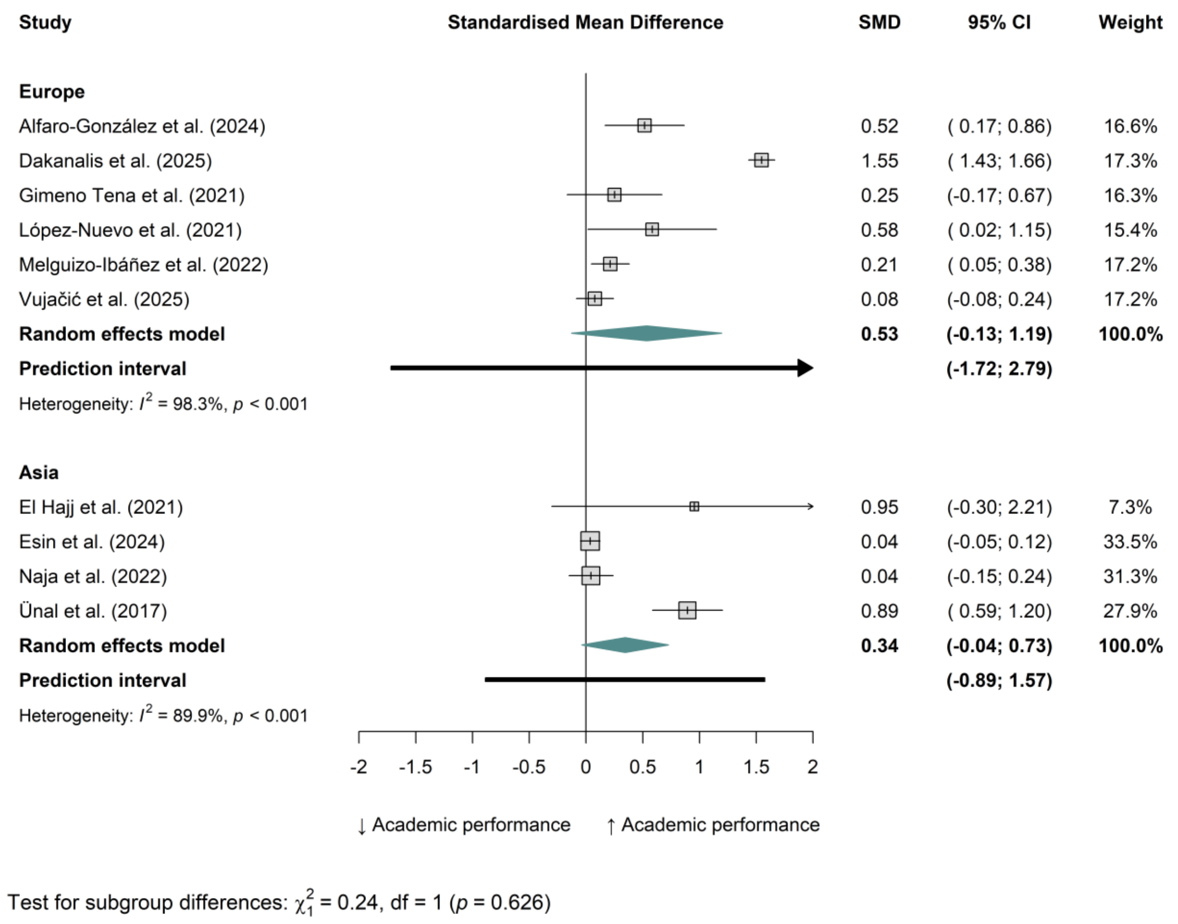

Figure S1. Subgroup analysis of the association between Mediterranean diet adherence and objective academic performance, stratified by geographic region (Europe vs. Asia). Random-effects forest plot of the standardized mean difference, with 95% confidence and prediction intervals per subgroup. ***Abbreviations:*** CI, confidence interval; SMD, standardized mean difference.

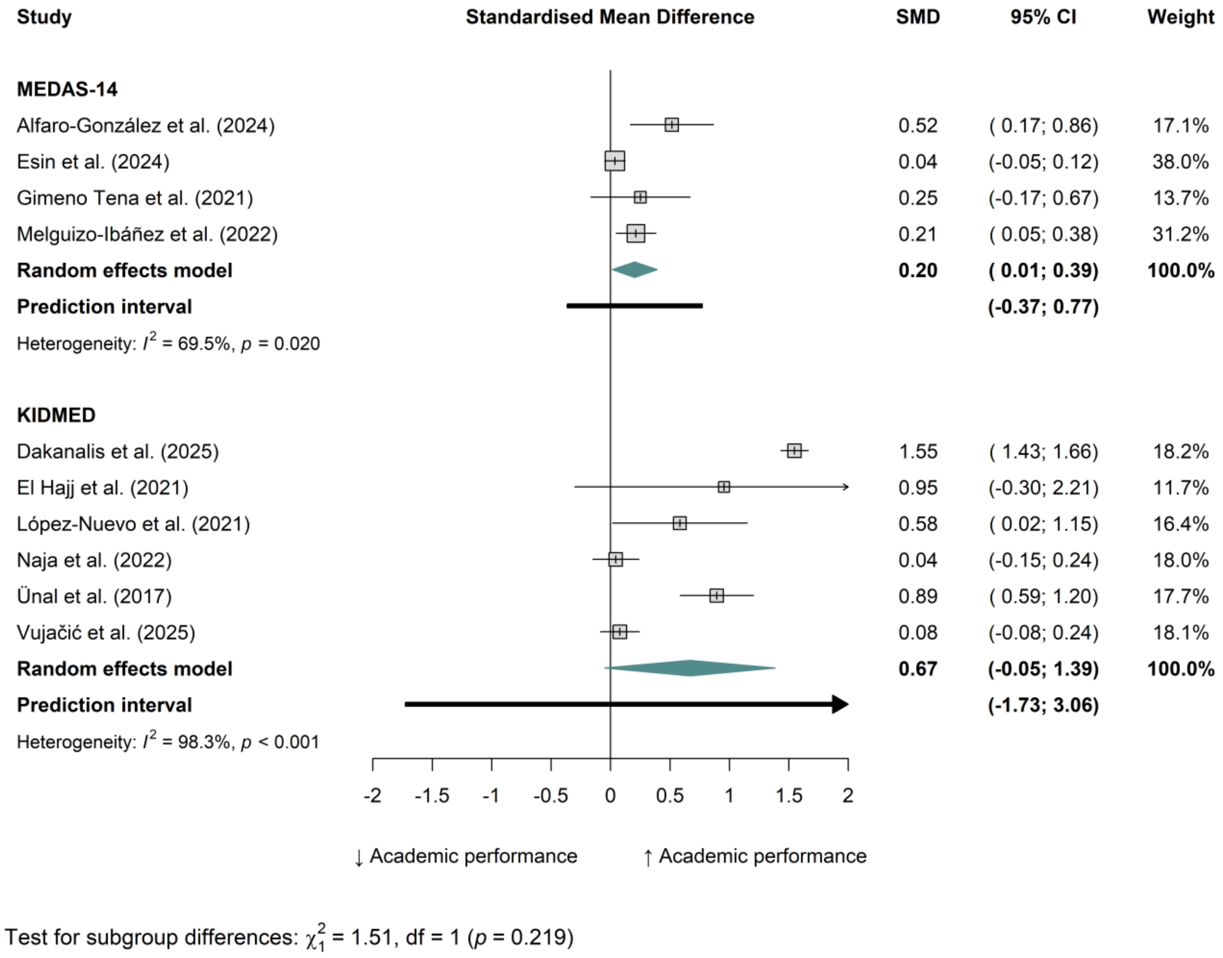

Figure S2. Subgroup analysis of the association between Mediterranean diet adherence and objective academic performance, stratified by Mediterranean diet adherence instrument (MEDAS-14 vs. KIDMED). Random-effects forest plot of the standardized mean difference, with 95% confidence and prediction intervals per subgroup. ***Abbreviations:*** CI, confidence interval; KIDMED, Mediterranean Diet Quality Index in Children and Adolescents; MEDAS, Mediterranean Diet Adherence Screener; SMD, standardized mean difference.

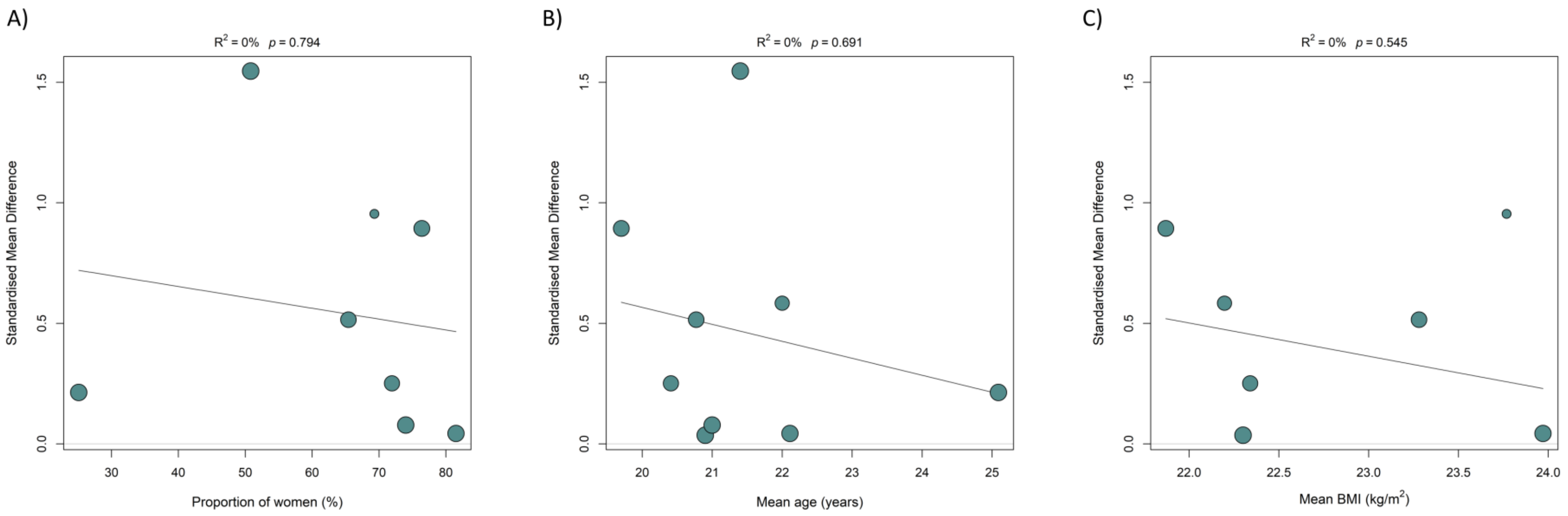

Figure S3. Random-effects meta-regression of the standardized mean difference for objective academic performance on three study-level moderators: **(A)** proportion of women (k = 8); **(B)** mean age (k = 9); and **(C)** mean body mass index (k = 7). Each bubble represents a study, sized proportionally to its weight; the solid line denotes the fitted regression slope. R^2 (proportion of between-study variance explained) and the p-value for each moderator are shown. ***Abbreviations:*** BMI, body mass index; SMD, standardized mean difference.

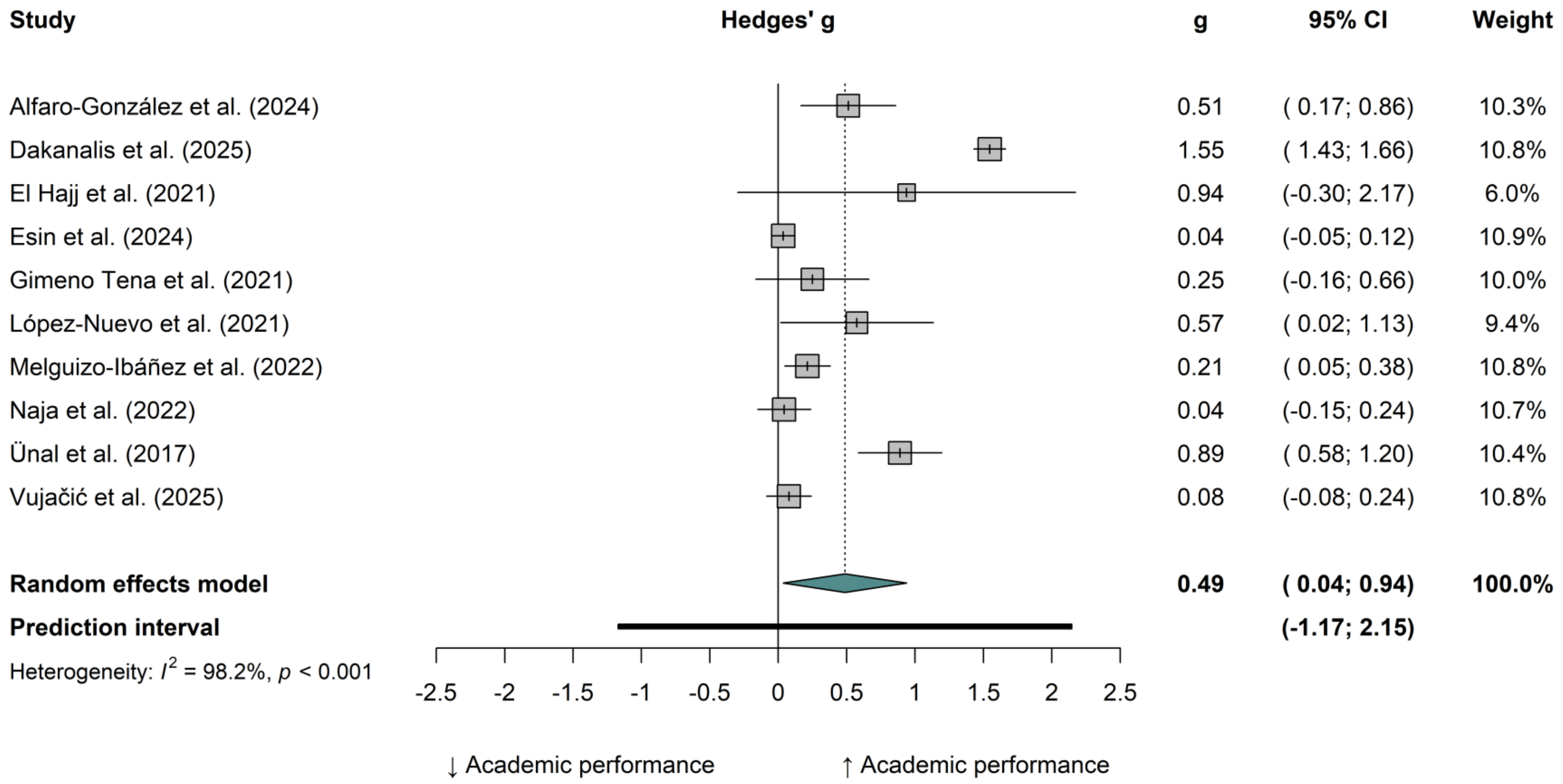

Figure S4. Sensitivity analysis of the primary meta-analysis re-estimated using Hedges' g (small-sample bias-corrected) instead of Cohen's d. Random-effects forest plot of the association between Mediterranean diet adherence and objective academic performance, with 95% confidence and prediction intervals. ***Abbreviations:*** CI, confidence interval; SMD, standardized mean difference.

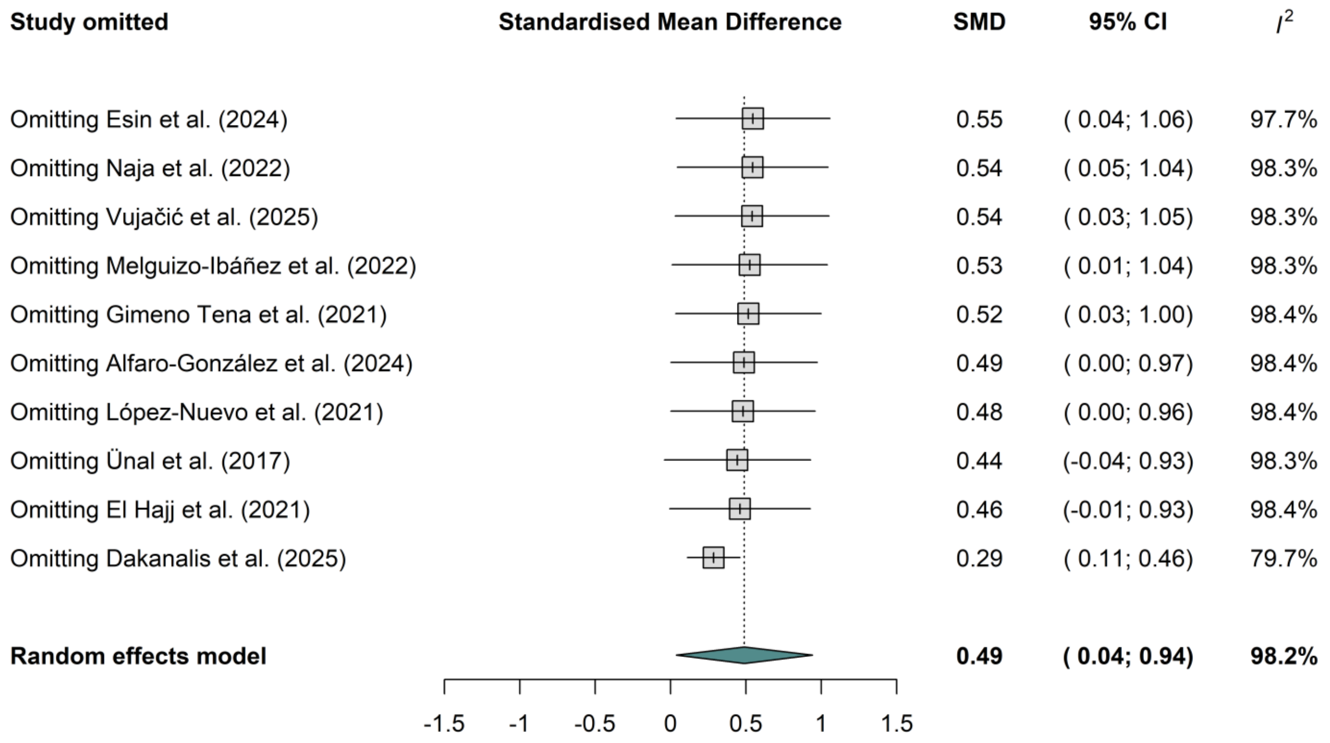

Figure S5. Leave-one-out sensitivity analysis of the primary meta-analysis (objective academic performance), showing the pooled random-effects estimate of the standardized mean difference recalculated after sequentially omitting each study. The 95% confidence interval and I^2 are reported for each iteration. ***Abbreviations:*** CI, confidence interval; I^2, statistical heterogeneity; SMD, standardized mean difference.

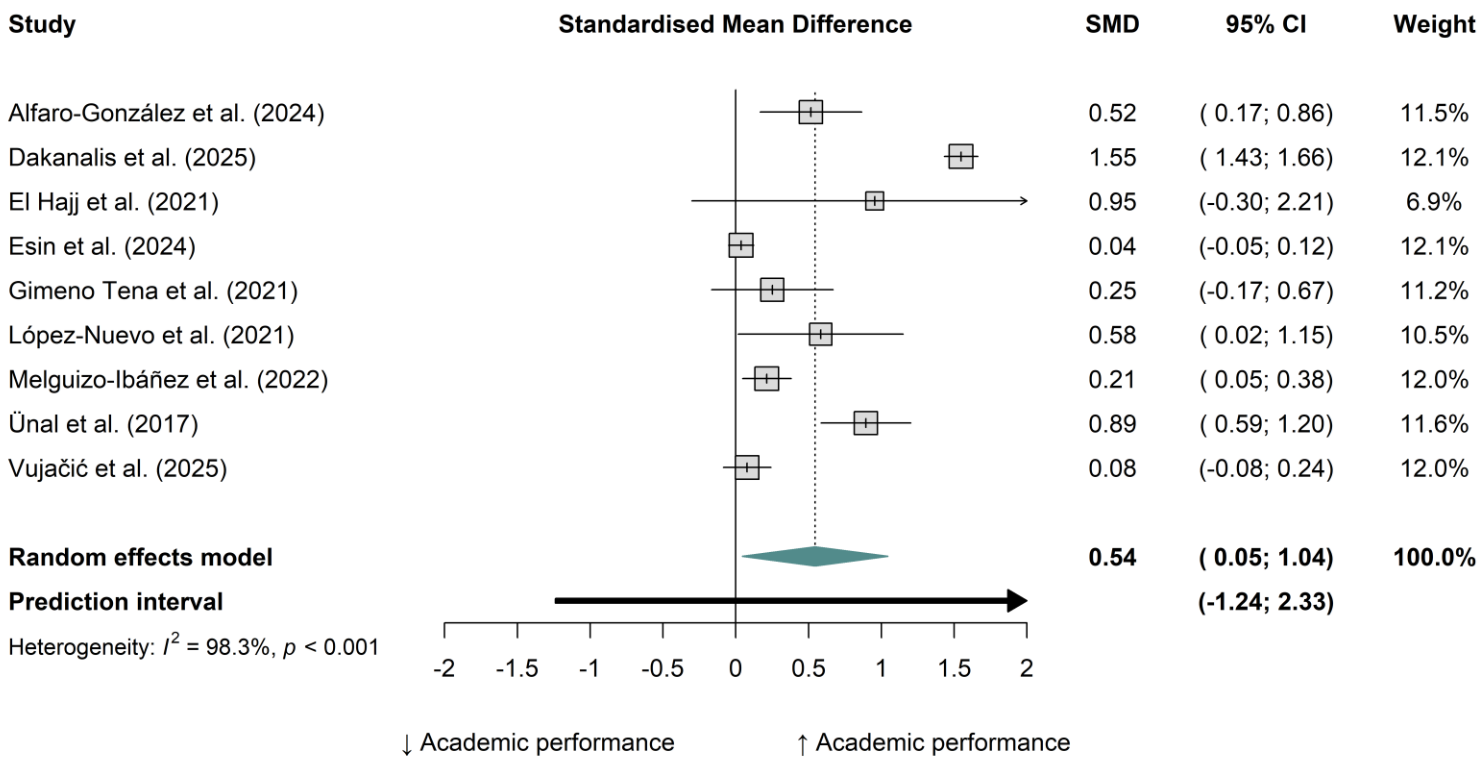

Figure S6. Sensitivity analysis of the primary meta-analysis (objective academic performance) restricted to studies conducted in Mediterranean countries, after excluding one non-Mediterranean study [11]. Random-effects forest plot of the standardized mean difference, with 95% confidence and prediction intervals. ***Abbreviations:*** CI, confidence interval; SMD, standardized mean difference.

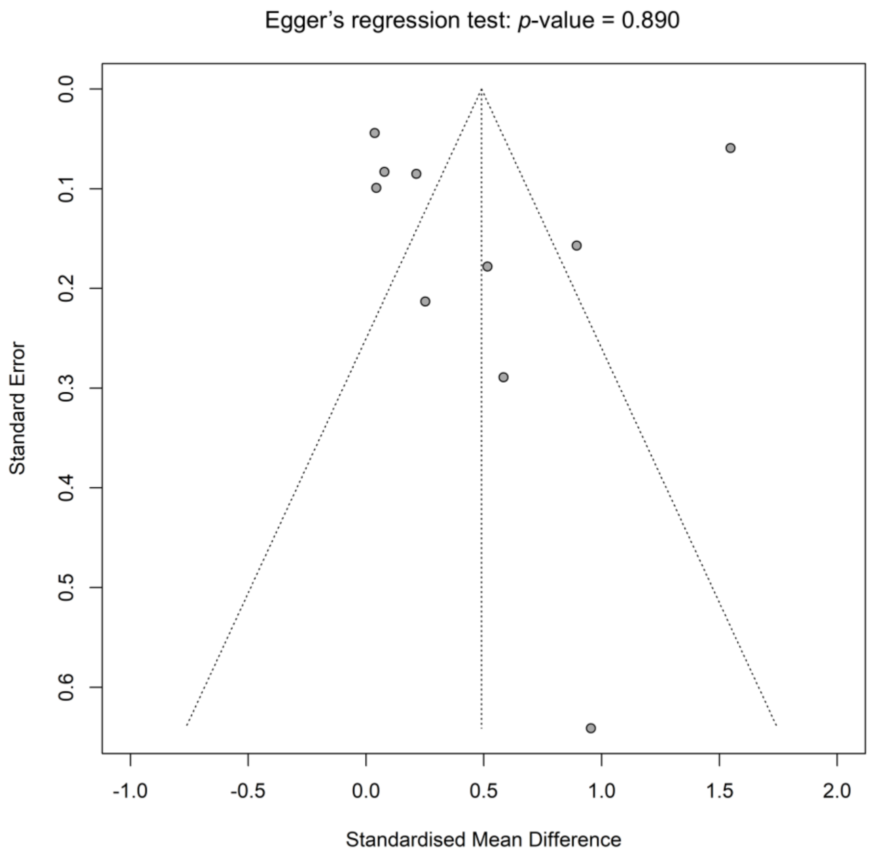

Figure S7. Funnel plot for the assessment of small-study effects and potential publication bias in the primary meta-analysis (objective academic performance), with the standardized mean difference plotted against its standard error. Funnel asymmetry was formally tested using Egger's regression test.

References

1. Page, M.J.; McKenzie, J.E.; Bossuyt, P.M.; Boutron, I.; Hoffmann, T.C.; Mulrow, C.D.; Shamseer, L.; Tetzlaff, J.M.; Akl, E.A.; Brennan, S.E.; et al. The PRISMA 2020 Statement: An Updated Guideline for Reporting Systematic Reviews. *BMJ* **2021**, n71, doi:10.1136/bmj.n71.
2. Stroup, D.F.; Berlin, J.A.; Morton, S.C.; Olkin, I.; Williamson, G.D.; Rennie, D.; Moher, D.; Becker, B.J.; Sipe, T.A.; Thacker, S.B.; et al. Meta-Analysis of Observational Studies in Epidemiology: A Proposal for Reporting. *JAMA* **2000**, *283*, 2008–2012, doi:10.1001/jama.283.15.2008.
3. Alfaro-González, S.; Garrido-Miguel, M.; Fernández-Rodríguez, R.; Mesas, A.E.; Bravo-Esteban, E.; López-Muñoz, P.; Rodríguez-Gutiérrez, E.; Martínez-Vizcaíno, V. Higher Adherence to the Mediterranean Diet Is Associated with Better Academic Achievement in Spanish University Students: A Multicenter Cross-Sectional Study. *Nutrition Research* **2024**, *126*, 193–203, doi:10.1016/j.nutres.2024.04.004.
4. Dakanalis, A.; Papadimitriou, K.; Alexatou, O.; Deligiannidou, G.-E.; Pappa, M.; Papadopoulou, S.K.; Louka, A.; Paschodimas, G.; Mentzelou, M.; Giaginis, C. Mediterranean Diet Compliance Is Related with Lower Prevalence of Perceived Stress and Poor Sleep Quality in University Students: A Cross-Sectional Study in Greece. *Nutrients* **2025**, *17*, 2174, doi:10.3390/nu17132174.
5. El Hajj, J.S.; Julien, S.G. Factors Associated with Adherence to the Mediterranean Diet and Dietary Habits among University Students in Lebanon. *Journal of Nutrition and Metabolism* **2021**, *2021*, 1–7, doi:10.1155/2021/6688462.
6. Esin, K.; Ayyıldız, F. Food Insecurity, Mediterranean Diet Adherence, and Psychosocial Health among University Students in Türkiye. *International Journal of Environmental Health Research* **2024**, *34*, 649–659, doi:10.1080/09603123.2023.2300405.
7. Gianfredi, V.; Nucci, D.; Tonzani, A.; Amodeo, R.; Benvenuti, A.L.; Villarini, M.; Moretti, M. Sleep Disorder, Mediterranean Diet and Learning Performance among Nursing Students: inSOMNIA, a Cross-Sectional Study. *Ann Ig* **2018**, *30*, 470–481, doi:10.7416/ai.2018.2258.
8. Gimeno Tena, A.; Esteve Clavero, A. Relación entre los hábitos saludables y el rendimiento académico en los estudiantes de la Universitat Jaume I. *Nutr Clín Diet Hosp* **2021**, *41*, doi:10.12873/412gimeno.
9. López-Nuevo, C.E.; Sanchez Molina, J.; Diaz Ureña, G. Adherence to Healthy Habits and Academic Performance in Vocational Education Students (Adherencia a Hábitos Saludables y Rendimiento Académico En Estudiantes de Formación Profesional). *Retos* **2021**, *42*, 118–125, doi:10.47197/retos.v42i0.87138.
10. Eduardo Melguizo Ibáñez; Félix Zurita Ortega; Georgian Badicu; José Luis Ubago Jiménez; Wilhelm Robert Grosz; Gabriel González Valero Adherence to the Mediterranean Diet and Its Association with Self-Concept and Academic and Physical Domain in Education Science Students: A Cross-Sectional Study. *JOMH* **2022**, *18*, 1, doi:10.31083/j.jomh1804100.
11. Naja, F.; Hasan, H.; Khadem, S.H.; Buanq, M.A.; Al-Mulla, H.K.; Aljassmi, A.K.; Faris, M.E. Adherence to the Mediterranean Diet and Its Association With Sleep Quality and Chronotype Among Youth: A Cross-Sectional Study. *Front. Nutr.* **2022**, *8*, 805955, doi:10.3389/fnut.2021.805955.
12. Ünal, G.; Uzdil, Z.; Kökdener, M.; Özenoğlu, A. Breakfast Habits and Diet Quality among University Students and Its Effect on Anthropometric Measurements and Academic Success. *Progress in Nutrition* **2017**, *19*, doi:10.23751/pn.v19i2.4900.
13. Vujačić, V.; Podovšovnik, E.; Planinc, S.; Krešić, G.; Kukanja, M. Bridging Knowledge and Adherence: A Cross-National Study of the Mediterranean Diet Among Tourism Students in Slovenia, Croatia, and Montenegro. *Sustainability* **2025**, *17*, 5440, doi:10.3390/su17125440.
14. Zurita-Ortega, F.; San Román-Mata, S.; Chacón-Cuberos, R.; Castro-Sánchez, M.; Muros, J. Adherence to the Mediterranean Diet Is Associated with Physical Activity, Self-Concept and Sociodemographic Factors in University Student. *Nutrients* **2018**, *10*, 966, doi:10.3390/nu10080966.

15. National Heart, Lung, and Blood Institute Quality Assessment Tool for Observational Cohort and Cross-Sectional Studies Available online: https://www.nhlbi.nih.gov/health-topics/study-quality-assessment-tools (accessed on 2 July 2026).
16. Guyatt, G.H.; Oxman, A.D.; Vist, G.E.; Kunz, R.; Falck-Ytter, Y.; Alonso-Coello, P.; Schünemann, H.J.; GRADE Working Group GRADE: An Emerging Consensus on Rating Quality of Evidence and Strength of Recommendations. *BMJ* **2008**, *336*, 924–926, doi:10.1136/bmj.39489.470347.AD.
17. *Cochrane Handbook for Systematic Reviews of Interventions*; Julian P. T. Higgins, Thomas, J., Chandler, J., Cumpston, M., Li, T., Page, M.J., Welch, V.A., Eds.; Cochrane book series; Second edition.; John Wiley & Sons: Chichester (UK), 2019; ISBN 978-1-119-53662-8.
18. Cohen, J. *Statistical Power Analysis for the Behavioral Sciences*; L. Erlbaum Associates, 1988.
